# Seeking adverse effects in systematic reviews of orthodontic interventions: a cross-sectional study (part 1)

**DOI:** 10.1186/s13643-023-02273-7

**Published:** 2023-07-03

**Authors:** Pauline A. J. Steegmans, Nicola Di Girolamo, Shandra Bipat, Reint A. Meursinge Reynders

**Affiliations:** 1 Department of Orthodontics, Academisch Centrum Tandheelkunde Amsterdam (ACTA), University of Amsterdam, Gustav Mahlerlaan 3004, Amsterdam, 1081 LA The Netherlands; 2 Department of Clinical Sciences, College of Veterinary Medicine, Cornell University, Ithaca, NY USA; 3 EBMVet, Via Sigismondo Trecchi 20, Cremona, CR 26100 Italy; 4 Department of Radiology and Nuclear Medicine, Amsterdam University Medical Center (Amsterdam UMC), Location AMC, Meibergdreef 9, Amsterdam, 1105 AZ The Netherlands; 5 Department of Oral and Maxillofacial Surgery, Amsterdam University Medical Center (Amsterdam UMC), Location AMC, Meibergdreef 9, Amsterdam, 1105 AZ The Netherlands; 6 Studio Di Ortodonzia, Via Matteo Bandello 15, Milan, 20123 Italy

**Keywords:** Orthodontics, Reporting, Systematic review, Interventions, Adverse effect, Adverse event, Harm, Safety, Side effect, Patient important outcomes

## Abstract

**Background:**

Systematic reviews that assess the benefits of interventions often do not completely capture all dimensions of the adverse effects. This cross-sectional study (part 1 of 2 studies) assessed whether adverse effects were sought, whether the findings on these effects were reported, and what types of adverse effects were identified in systematic reviews of orthodontic interventions.

**Methods:**

Systematic reviews of orthodontic interventions on human patients of any health status, sex, age, and demographics, and socio-economic status, in any type of setting assessing any type of adverse effect scored at any endpoint or timing were eligible. The Cochrane Database of Systematic Reviews and 5 leading orthodontic journals were manually searched for eligible reviews between August 1 2009 and July 31 2021. Study selection and data extraction was conducted by two researchers independently. Prevalence proportions were calculated for four outcomes on seeking and reporting of adverse effects of orthodontic interventions. Univariable logistic regression models were used to determine the association between each one of these outcomes and the journal in which the systematic review was published using the eligible Cochrane reviews as reference.

**Results:**

Ninety-eight eligible systematic reviews were identified. 35.7% (35/98) of reviews defined seeking of adverse effects as a research objective, 85.7% (84/98) sought adverse effects, 84.7% (83/98) reported findings related to adverse effects, and 90.8% (89/98) considered or discussed potential adverse effects in the review. Reviews in the journal Orthodontics and Craniofacial Research compared with Cochrane reviews had approximately 7 times the odds (OR 7.20, 95% CI 1.08 to 47.96) to define seeking of adverse effects in the research objectives. Five of the 12 categories of adverse effects accounted for 83.1% (162/195) of all adverse effects sought and reported.

**Conclusions:**

Although the majority of included reviews sought and reported adverse effects of orthodontic interventions, end-users of these reviews should beware that these findings do not give the complete spectrum on these effects and that they could be jeopardized by the risk of non-systematically assessing and reporting of adverse effects in these reviews and in the primary studies that feed them. Much research is ahead such as developing core outcome sets on adverse effects of interventions for both primary studies and systematic reviews.

**Supplementary Information:**

The online version contains supplementary material available at 10.1186/s13643-023-02273-7.

## Background

To get a balanced perspective of an intervention, systematic reviewers need to report both its beneficial and adverse effects [1]. In this cross-sectional study we assessed whether adverse effects were sought, whether the findings on these effects were reported, and what types of adverse effects were identified in systematic reviews published in the Cochrane Database of Systematic reviews [2] and in 5 leading orthodontic journals.

‘Cochrane defines an adverse effect as ‘an adverse event for which the causal relation between the intervention and the event is at least a reasonable possibility’ [3, 4]. This definition and other key terms used in this manuscript are listed in Table 1 [5, 6]. A wide body of epidemiological studies has shown that adverse effects of interventions in primary research studies are often under-assessed, and/or under-reported, and/or distorted [7–13]. These issues can misinform anyone trying to make valid decisions on a healthcare intervention. An extension of the Consolidated Standards of Reporting Trials (CONSORT) Statement was developed to tackle poor reporting of harms in randomized trials [14]. Since the publication of this statement, the reporting of adverse events in clinical trials has improved, but is still suboptimal [10, 12, 15, 16].

**Table 1:** Glossary of terms

Term	Definition
Systematic review	Cochrane [5] defines a systematic review as follows: ‘A systematic review attempts to identify, appraise and synthesize all the empirical evidence that meets pre-specified eligibility criteria to answer a specific research question. Researchers conducting systematic reviews use explicit, systematic methods that are selected with a view aimed at minimizing bias, to produce more reliable findings to inform decision making.’
Intervention review	Cochrane [5] defines an intervention review as follows: ‘Intervention reviews assess the benefits and harms of interventions used in healthcare and health policy.’
Orthodontic interventions	Steegmans et al. [6] defined orthodontic interventions as follows: ‘Orthodontic interventions refer to the use of any type of orthodontic appliance to move teeth or change the jaw size or position for orthodontic purposes. These interventions also include appliances to maintain or stabilize the results of orthodontic treatment, for example retainers.’
Adverse effect	Cochrane [3, 4], defines an adverse effect as ‘an adverse event for which the causal relation between the intervention and the event is at least a reasonable possibility’

Systematic reviews could provide even more information on adverse effects, because they assess large amounts of data from a wide spectrum of sources (possibly including both published and unpublished data). By assessing the data of multiple single studies, systematic reviewers can make a more balanced assessment of an intervention. This is an important issue, because serious adverse effects may occur rarely and might be missed in single studies. However, epidemiological research showed that the seeking and reporting of adverse effects of interventions and the methods used to identify and synthesize them [17–21] were also poor in systematic reviews. The Preferred Reporting Items for Systematic reviews and Meta-Analyses (PRISMA) harms checklist was published in 2016 [22] to improve harms reporting in systematic reviews, but its consequences are still largely unknown.

We performed 2 cross-sectional studies on assessing and reporting of adverse effects in systematic reviews of orthodontic interventions. In this study (part 1), we assessed whether adverse effects were sought and reported and what findings on these adverse effects were reported in systematic reviews of orthodontic interventions published in the Cochrane Database of Systematic reviews [2] and in 5 leading orthodontic journals. In a second study (part 2) we assessed the reporting on adverse effects and the presence of spin on adverse effects in the abstracts of these reviews [23]. Adverse effects of orthodontic interventions refer to for example, pain associated with orthodontic tooth movement, root resorption, decalcifications, periodontal problems, relapse, and undesired health experiences [24]. Recent (November 22 2021) scoping searches confirmed that our research objectives have not been addressed previously.

## Objectives

The objectives of this research study are formulated in the following four research questions:

1. Was seeking of adverse effects of interventions defined as a research objective of the review?
2. Did the review seek any findings related to adverse effects of interventions in the included studies?
3. Did the review report findings related to adverse effects of interventions sought in the included studies?
4. Were potential adverse effects of the intervention considered, discussed (weighed) anywhere in the review?

We also assessed what adverse effects of interventions were defined as research objectives and what adverse effects of interventions were sought and reported in the review.

## Methods

The Strengthening the Reporting of Observational Studies in Epidemiology (STROBE) statement [25] and the PRISMA 2020 statement [26, 27] were consulted for reporting this cross-sectional study. The STROBE checklist of items for reporting cross-sectional studies was presented in Additional file 1. The methods for this cross-sectional study were explained in our published protocol [6] and can be consulted through the following link https://systematicreviewsjournal.biomedcentral.com/articles/10.1186/s13643-019-1000-1. We adopted the framework of this protocol to report the methods section of this study and its additional files. Raw data are recorded in Open Science Framework (https://osf.io/ka7mp/). Differences between methods originally planned in the protocol and those implemented in the final research study were given with the rationales for these differences in Additional file 2. No patients were involved in the development of the protocol or in the conduct of this study.

### Eligibility criteria

The eligibility criteria have been published previously [6, 28] and are presented again in Table 2 [29].

**Table 2:** Eligibility criteria

Item	Included	Excluded
Study designs	Systematic reviews of orthodontic interventions. The definition of systematic review, intervention review, and orthodontic interventions listed in the Glossary of terms will be used to assess whether a review is eligible (Table 1)	1) Non-interventional reviews such as, ‘Methodology’. ‘Diagnostic’, ‘Qualitative’, ‘Prognostic’ etc 2) Rapid and scoping reviews 3) Systematic reviews with Bayesian network meta-analysis 5) Systematic reviews of interventions that did not find any eligible studies (empty reviews)
Participants	Systematic reviews on any type of patients undergoing orthodontic interventions, i.e., patients of any health status, sex, age, and demographics, and socio-economic status	1) Intervention reviews that focus exclusively on patients with congenital anomalies, for example with cleft lip and palate 2) Systematic reviews of animal or laboratory studies
Interventions	1) Systematic reviews that assessed the effects of clinical orthodontic interventions. Clinical orthodontic interventions refer to the use of any type of orthodontic appliance that are used to move teeth or change the jaw size or position for orthodontic purposes 2) Systematic reviews of interventions with appliances to maintain or stabilize the outcomes of orthodontic treatment, for example retainers 3) Systematic reviews of orthodontic interventions that compared the effects of orthodontic treatment with or without additional interventions such as pharmacological or small surgical interventions, e.g., periodontal or implant surgery 4) No exclusion criteria were applied to the characteristics of the operator who conducted the interventions	1) Systematic reviews in which patients receive orthodontic treatment, but in which the effects of other interventions, e.g., periodontal surgery, were compared and not the effects of orthodontic interventions 2) Systematic reviews of interventions in which orthodontic appliances were specifically used for other purposes, e.g., changing jaw positions to treat respiration or temporomandibular disorders 3) Systematic review of orthodontic interventions that included orthognathic surgery 4) Systematic reviews that focussed exclusively on adverse effects of interventions 5) Systematic reviews that did not assess a specific orthodontic intervention, but referred to orthodontic treatment as a whole
Outcomes	1) Any adverse effect of orthodontic interventions scored at any endpoint or timing 2) The effects of orthodontic interventions did not refer just to outcomes related to tooth and jaw size and positions, but also to broader outcomes such as periodontal health, esthetic changes, the health of the temporomandibular joint, patient health experiences, and economic issues associated with the interventions 3) The reporting of outcomes on adverse effects did not determine eligibility of reviews for this cross-sectional study, i.e., reviews were not excluded because they did not report measured outcome data in a ‘usable’ way [29]	No exclusion criteria
Stetting	Any type of setting in which the interventions were conducted, i.e., university or private practice	No exclusion criteria

### Information sources and search strategy

The information sources for this study were the Cochrane Database of Systematic Reviews [2] and the websites of 5 leading orthodontic journals. The selection of these 5 orthodontic journals was based on having been published at least 10 years and the highest impact factor [30]. The impact factor in 2018, i.e., the year when the protocol was developed, was used to select these journals. The 5 selected orthodontic journals are: European Journal of Orthodontics [EJO], American Journal of Orthodontics and Dentofacial Orthopedics [AJODO], Angle Orthodontist (AO), The Korean Journal of Orthodontics (KJO), and Orthodontics and Craniofacial Research (O&CR). The impact factors of these journals are listed in Additional file 2. August 1 2009 was chosen as the inception date for searching the information sources, because it coincides with the publication of the PRISMA statement and guidance document on 21 July 2009 [31, 32]. Eligible systematic reviews were manually searched in these information sources from the inception date until July 31 2021.

### Study records

#### Data management

All study selection and data extraction procedures were conducted independently by 2 authors (PS and RMR). Pilot tests were done a priori to train and calibrate these operators [33]. Disagreements between these reviewers during these study selection and data collection were resolved in the following order: Firstly, through discussions; secondly, through rereading the article in question; and thirdly, through contacting of the authors of the pertinent manuscript by email to obtain additional information that could help with decision-making [27]. Persistent disagreements were resolved through discussions with a methodologist (SB). All eligible systematic reviews with their supplementary files were downloaded as PDFs and merged in binder files [34, 35]. Data were collected in an Excel spreadsheet [36].

#### Study selection and data collection procedures

Titles and abstracts were screened for eligible reviews in the websites of the 5 selected orthodontic journals. Eligible Cochrane reviews were searched in the ‘Dentistry and Oral health’ section of the Cochrane Database of Systematic Reviews [2]. When Cochrane reviews were updated, we only considered the latest published version. A PRISMA flow diagram was presented to illustrate the selection process of the eligible reviews [26, 27]. All included studies and excluded studies were reported and the rationales for exclusion were given in Additional file 3. Contacting of authors was not necessary to clarify eligibility or data extraction issues. We used our pilot tested data collection forms for the extraction of all pertinent data items. These forms are presented in Additional file 2. The entire eligible review except the abstract and protocol were searched, i.e., the main text, tables, figures, and supplemental files. This strategy was implemented for all eligible reviews. In Cochrane systematic reviews, we also did not search data items in the plain language summary.

### Assigning adverse effects of orthodontic interventions

Cochrane defines an adverse effect as ‘an adverse event for which the causal relation between the intervention and the event is at least a reasonable possibility’ [3, 4]. These events can have a permanent or temporary adverse effect on the health condition of the patient. Root resorption, decalcifications of enamel or caries around orthodontic appliances are well known permanent adverse effects of orthodontic interventions, while pain and discomfort during tooth movement are generally temporary adverse effects. Events associated with orthodontic interventions that could have an adverse effect on the health condition were also labeled as adverse events, e.g., breaking of appliances, failure to complete treatment, and tolerability of orthodontic appliances.

According to our protocol we adopted the framework of known orthodontic adverse effects as reported previously by Preoteasa et al. [24] (Additional file 2) and made some changes in labeling the headings of the various categories of adverse effects (Additional file 2). A total of 12 categories of adverse effects were defined. Additional adverse effects identified during our data extraction procedures were also included in this framework and when ambiguous the rationale for including these adverse effects was given. The following types of adverse events were not labeled as adverse effects: (1) effects that do not refer to health conditions and could be ambiguous, e.g., costs, duration of treatment, number of appointments etc. (2) effects that refer to pre-existing health problems that can actually improve as a result of the intervention, e.g., respiratory problems as a result of maxillary expansion or self-esteem as a result of the retraction of protruding maxillary incisors.

### Power calculation

Epitools epidemiological software was used to calculate the required sample size of eligible systematic reviews of orthodontic interventions [37]. We calculated the required sample size of 73 reviews based on the following input: estimated proportion 0.25, desired precision 0.1, and confidence level 0.95. The estimated proportion was based on the findings in our pilot tests as reported in our protocol [6]. These pilot test showed that findings related to adverse effects were sought in 3 of 12 systematic reviews on orthodontic interventions representing the estimated proportion of 0.25 (3/12).

### Outcomes and statistical analyses

We reported the number of retrieved systematic reviews and eligible reviews and calculated the prevalence proportions that addressed our research questions. All outcomes were calculated as originally planned in our published protocol [6]. Prevalence proportions were calculated for: (1) all journals together (2) each journal separately and (3) the group of 5 leading orthodontic journals together and the Cochrane reviews separately. Univariable logistic regression models were built to determine the association between each one of four outcomes and the journal in which the systematic review was published, using the Cochrane Database of Systematic reviews as reference. The strength of association was quantified using odds ratios (OR), and 95% confidence intervals (95% CI). Analyses were performed with the use of commercial software (IBM SPSS 22.0, SPSS Inc, Chicago, IL). A two-sided *P* value of 0.05 was considered to be statistically significant.

## Results

### Results of the search

**Fig. 1:**
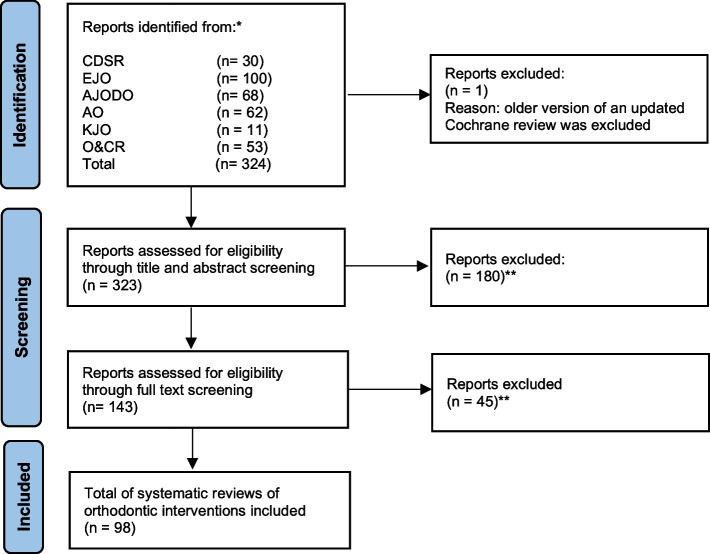
Flow diagram for the selection of systematic reviews of orthodontic interventions

Through our searches in the databases of the Cochrane Database of Systematic reviews and the 5 leading orthodontic journals we identified 324 reports. One Cochrane review was excluded, because it was later updated leaving 323 reports for screening. A total of 180 papers was excluded during the title and abstract screening and 45 during full text screening. A total of 98 systematic reviews fulfilled the eligibility criteria of this study. The results of the individual selection steps are presented in a PRISMA flow diagram (Fig. 1) [26, 27]. All included studies are listed in Additional file 3 and excluded studies with the rationale for their exclusion are given in Additional file 4.

### Included studies

**Fig. 2:**
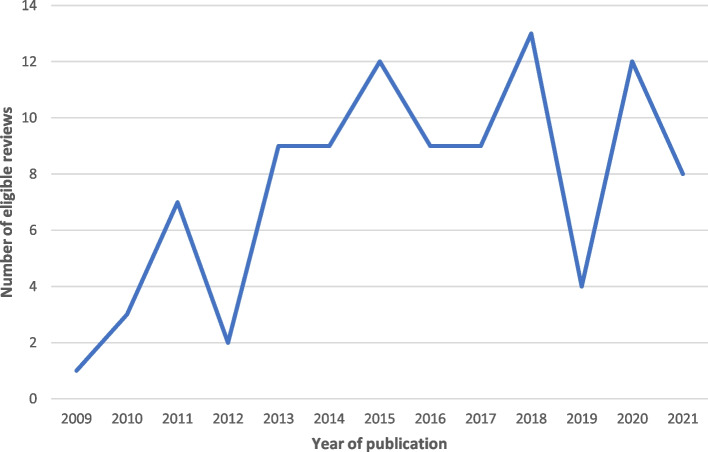
Line chart of the number of eligible systematic reviews published between August 1 2009 and July 31 2021

Figure 2 presents the number of eligible systematic reviews of orthodontic interventions published during the eligible time span. Table 3 presents the number of eligible reviews for each selected journal and shows that 72.4% (71/98) of the included reviews came from the EJO, AJODO, and AO. Table 3 also gives the types of orthodontic interventions for each of these journals, which are divided in three categories. Category 1 refers to orthodontic interventions to move teeth modify jaws such as fixed orthodontic appliances or palatal expansion appliances. Category 2 refers to orthodontic interventions that also include additional surgical, pharmacological or vibrational interventions such as mini-implants, prostaglandins, piezo surgery, or vibratory stimulation. Category 3 refers to orthodontic interventions with appliances to maintain or stabilize orthodontic treatment results such as retainers. The majority of included reviews, 70.4% (69/98), assessed orthodontic interventions to move teeth or modify jaws and 28.6% (28/98) assessed orthodontic interventions with additional surgical, pharmacological or vibratory interventions.

**Table 3:** Characteristics of included reviews

Type of orthodontic intervention	Cochrane	EJO	AJODO	AO	KJO	O&CR	Total
Category 1. Orthodontic interventions to move teeth or modify jaws	7	21	14	15	2	10	69
Category 2. Orthodontic interventions with additional surgical, pharmacological or vibratory interventions	3	6	6	8	1	4	28
Category 3. Orthodontic interventions to maintain or stabilize orthodontic results	0	1	0	0		0	1
Total	10	28	20	23	3	14	98

### Outcomes to the research questions

**Fig. 3:**
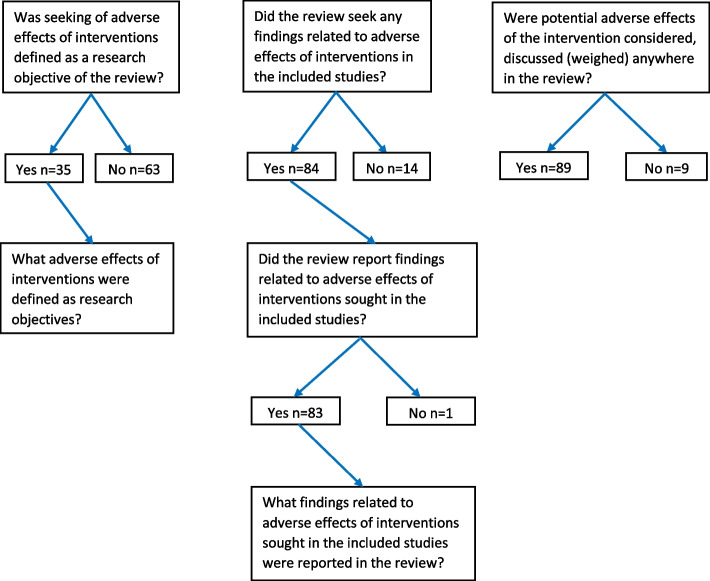
Seeking adverse effects in systematic reviews of orthodontic interventions

Figure 3 presents the answers to each individual research question and Table 4 gives the proportions. We reported the proportions in answering the four research questions over time in Table 5. The prevalence of reviews that defined seeking of adverse effects of interventions as a research objective was low, i.e., 35.7% in the 98 eligible reviews. Instead, the proportions that addressed the other 3 research question were 85% and higher indicating that seeking and reporting of findings related to adverse effects of interventions in the included studies and considering or discussing potential adverse effects anywhere in the review were implemented in most of the eligible reviews. As compared to the Cochrane Database of Systematic Reviews, the journal of Orthodontics and Craniofacial research had approximately 7 times the odds (OR 7.20, 95%CI 1.08 to 47.96) to report that adverse effects were sought in the research objectives. The other journals were not significantly more likely to report that adverse effects were sought in the research objectives (Table 6). For the other 3 outcomes, no statistical analysis was performed considering the low variability in the response scored (prevalence of ‘no’ ranging from 9.2 to 15.3%) and the overall small sample sizes (Table 4).

**Table 4:** Outcomes on seeking adverse effects in systematic reviews of orthodontic interventions

Description of outcomes	Cochrane	EJO	AJODO	AO	KJO	O&CR	All orthodontic journals	All journals
The number of identified systematic reviews	29	100	68	61	11	53	293	322
The number of eligible systematic reviews of orthodontic interventions	10	28	20	23	3	14	88	98
The prevalence of eligible systematic reviews of orthodontic interventions that defined seeking of adverse effects of interventions as a research objective of the review^a^	20.0% (2/10)	32.1% (9/28)	45% (9/20)	21.7 (5/23)	33.3% (1/3)	64.3% (9/14)	47.7% (33/88)	35.7% (35/98)
The prevalence of eligible systematic reviews of orthodontic interventions that sought any findings related to adverse effects of interventions in the included studies^a^	100.0% (10/10)	92.9% (26/28)	75.0% (15/20)	78.3% (18/23)	100.0% (3/3)	85.7% (12/14)	84.1% (74/88)	85.7% (84/98)
The prevalence of eligible systematic reviews of orthodontic interventions that reported findings related to the adverse effects of interventions sought in the included studies^a^	100.0% (10/10)	92.9% (26/28)	70.0% (14/20)	78.3% (18/23)	100.0% (3/3)	85.7% (12/14)	83.0% (73/88)	84.7% (83/98)
The prevalence of eligible systematic reviews of orthodontic interventions that considered, discussed (weighed) potential adverse effects of the intervention anywhere in the review ^a^	100.0% (10/10)	92.9% (26/28)	85.0% (17/20)	91.3% (21/23)	100.0% (3/3)	85.7% (12/14)	89.8% (79/88)	90.8% (89/98)

^a^The denominator for calculating the proportions for each journal is the number of included reviews for that journal. The denominator for calculating the proportions for all journals together is the total of included reviews, i.e., 98

**Table 5:** Outcomes on seeking adverse effects in systematic reviews of orthodontic interventions by year of publication

		Was seeking of adverse effects of interventions defined as a research objective of the review		Did the review seek any findings related to adverse effects of interventions in the included studies?		Did the review report findings related to adverse effects of interventions sought in the included studies?		Were potential adverse effects of the intervention considered, discussed (weighed) anywhere in the review?		
Year		Yes	No	Yes	No	Yes	No	Yes	No	Total
2009	Count	0	1	1	0	1	0	1	0	1
	% within Year	0.0%	100.0%	100.0%	0.0%	100.0%	0.0%	100.0%	0.0%	100.0%
2010	Count	1	2	2	1	2	1	3	0	3
	% within Year	33.3%	66.7%	66.7%	33.3%	66.7%	33.3%	100.0%	0.0%	100.0%
2011	Count	3	4	6	1	6	1	6	1	7
	% within Year	42.9%	57.1%	85.7%	14.3%	85.7%	14.3%	85.7%	14.3%	100.0%
2012	Count	2	0	2	0	2	0	2	0	2
	% within Year	100.0%	0.0%	100.0%	0.0%	100.0%	0.0%	100.0%	0.0%	100.0%
2013	Count	3	6	9	0	9	0	9	0	9
	% within Year	33.3%	66.7%	100.0%	0.0%	100.0%	0.0%	100.0%	0.0%	100.0%
2014	Count	3	6	7	2	7	2	7	2	9
	% within Year	33.3%	66.7%	77.8%	22.2%	77.8%	22.2%	77.8%	22.2%	100.0%
2015	Count	3	9	9	3	9	3	12	0	12
	% within Year	25.0%	75.0%	75.0%	25.0%	75.0%	25.0%	100.0%	0.0%	100.0%
2016	Count	1	8	6	3	6	3	7	2	9
	% within Year	11.1%	88.9%	66.7%	33.3%	66.7%	33.3%	77.8%	22.2%	100.0%
2017	Count	2	7	8	1	7	2	8	1	9
	% within Year	22.2%	77.8%	88.9%	11.1%	77.8%	22.2%	88.9%	11.1%	100.0%
2018	Count	4	9	10	3	10	3	10	3	13
	% within Year	30.8%	69.2%	76.9%	23.1%	76.9%	23.1%	76.9%	23.1%	100.0%
2019	Count	3	1	4	0	4	0	4	0	4
	% within Year	75.0%	25.0%	100.0%	0.0%	100.0%	0.0%	100.0%	0.0%	100.0%
2020	Count	7	5	12	0	12	0	12	0	12
	% within Year	58.3%	41.7%	100.0%	0.0%	100.0%	0.0%	100.0%	0.0%	100.0%
2021	Count	3	5	8	0	8	0	8	0	8
	% within Year	37.5%	62.5%	100.0%	0.0%	100.0%	0.0%	100.0%	0.0%	100.0%
Total	Count	35	63	84	14	83	15	89	9	98
	% within Year	35.7%	64.3%	85.7%	14.3%	84.7%	15.3%	90.8%	9.2%	100.0%

**Table 6:** Results of univariable logistic regression including journal as a predictor variable of seeking adverse effects as an objective of the systematic review

Journal	Odds ratio	Lower 95% CI	Upper 95% CI	*P* value
Cochrane	1	–	–	–
AJODO	3.27	0.55	19.45	0.19
AO	1.11	0.18	6.99	0.91
EJO	1.90	0.33	10.80	0.47
KJO	2.00	0.12	34.82	0.63
O&C	7.20	1.08	47.96	0.04

### Labeling adverse effects of orthodontic interventions

The type of adverse effects most frequently defined as research objectives were adverse effects related to (1) tooth structures, (2) periodontal tissues, (3) undesired treatment results, (4) relapse and stability, and (5) negative qualitative experiences by the patient or carer(s) (Table 7). These were also the most prevalent types of adverse effects sought in the included studies and reported in the review and accounted for 83.1% (162/195) of all adverse effects sought and reported (Table 8). We were able to categorize all 195 adverse effects except one and labeled it ‘Additional adverse effects’ (Table 8).

**Table 7:** Type of adverse effects defined as research objectives of the review

Adverse effects related to	Prevalence
Tooth structures	16.7% (8/48)
Periodontal tissues	12.5% (6/48)
Intraoral (non-tooth or periodontal) tissues	0.0% (0/48)
Extraoral tissues (non-temporomandibular tissues)	0.0% (0/48)
Temporomandibular tissues and disorders	2.1% (1/48)
Undesired treatment results	18.8% (9/48)
Relapse and stability	20.8% (10/48)
Negative qualitative experiences by the patient or carer(s)	18.8% (9/48)
Appliance failure	0.0% (0/48)
Gastro-intestinal	0.0% (0/48)
Non-defined	10.4% (5/48)
Additional adverse effects	0.0% (0/48)

**Table 8:** Type of adverse effects sought and reported in the review

Adverse effects related to	Prevalence
Tooth structures	12.8% (25/195)
Periodontal tissues	13.8% (27/195)
Intraoral (non-tooth or periodontal) tissues	2.6% (5/195)
Extraoral tissues (non-temporomandibular tissues)	0.5%(1/195)
Temporomandibular tissues and disorders	4.1% (8/195)
Undesired treatment results	22.1% (43/195)
Relapse and stability	18.5% (36/195)
Negative qualitative experiences by the patient or carer(s)	15.9% (31/195)
Appliance failure	7.7% (15/195)
Gastro-intestinal	0.5%(1/195)
Non-defined	1.0% (2/195)
Additional adverse effects^a^	0.5%(1/195)

^a^General disorders, injury, poisoning and procedural complications, musculoskeletal and connective tissue disorders, nervous system disorders and respiratory, thoracic and mediastinal disorders, safety of the adjunctive intervention

## Discussion

### Principal findings of the study

This cross-sectional study showed that in 35.7% (35/98) of reviews of orthodontic interventions seeking of adverse effects was defined as an objective. In 85.7% (84/98) of these reviews, findings related to adverse effects of interventions were sought and in 84.7% (83/98) the reviewers reported on these findings. In more than 90% (89/98) of included systematic reviews, the reviewers discussed (weighed) potential adverse effects of interventions somewhere in the review. Five types of adverse effects accounted for 83.1% (162/195) of adverse effects that were sought and reported in the eligible reviews.

### Comparisons with other studies

The proportion of included reviews that defined seeking of adverse effects as a research objective was low, i.e., 35.7% (35/98) in both Cochrane and non-Cochrane systematic reviews (Table 4). Assessing potential adverse effects of interventions is considered a mandatory item when setting the research question for Cochrane intervention reviews [1]. Not defining seeking of adverse effects as a research objective can mislead end-users of systematic reviews. Authors therefore need to include this item in their research objectives and editors and peer reviewers should verify its implementation.

The proportions of reviews that reported findings related to adverse effects of interventions were higher in this sample of orthodontic reviews (84.7% (83/98) compared with gastroenterology reviews (66.7% (52/78) [18], Cochrane reviews of interventions (75.6% (59/78), and Database of Abstracts of Reviews of Effects (DAREs) reviews (48.1% (38/79) [38]. Explanations for these higher proportions could be: (1) the time period of inclusion of reviews (2) the research design and type of interventions of the studies included in the reviews (3) the field of research. Orthodontic research could be more focused on assessing adverse effects of interventions than other fields, because this assessment is an integral part of routine clinical practice. For example, assessing adverse effects such as undesired treatment results and relapse and stability are part of everyday problems in orthodontic practice and accounted for 40.5% (79/195) of adverse effects sought and reported in this sample of systematic reviews of orthodontic interventions (Table 8).

#### Strengths and limitations

This cross-sectional study has the following strengths: (1) scoping searches were conducted to identify knowledge gaps, (2) pilot studies were conducted to calibrate researchers and fine-tune research questions and methodology, (3) a protocol was developed and published a priori [6], and (4) all raw data were included with this manuscript or recorded in Open Science Framework (https://osf.io/ka7mp/). This study also has limitations. First, the findings of this cross-sectional study are expected to be better than those reported in the entire body of orthodontic literature, because we assessed reviews published in the five leading orthodontic journals and those listed in the Cochrane Database of Systematic Reviews. Second, the risk of selective (non) reporting bias regarding adverse effects in the eligible reviews. Third, only reviews published in a pre-established period (August 1 2009 until July 31 2021) were eligible, instead of having considered a larger sample, e.g., by having included reviews prior to the inception date. However, we chose this inception date, because it coincides with the launch of the PRISMA statement [31, 32], which provides reviewers better guidance on reporting.

#### Implications and future research

Several of our findings seem promising at a first glance. For example, the proportion of reviews that sought and reported adverse effects was relatively high, i.e., (84.7% (83/98), but a variety of issues has to be considered when interpreting this finding. First, this proportion only refers to whether or not reviewers implemented this item, but not how. For example, the reviewers could have reported on just one or a selection of all adverse effects assessed and reported in the eligible studies for their reviews. Second, this proportion also does not give any information on the magnitude, and duration of adverse effects nor on the time points for assessing them. Third, we do not know whether all adverse effects were indeed sought and reported as originally planned in the registered protocols of the included reviews. For example, Parsons et al. [39] showed that this was not the case in their sample of systematic reviews of health care interventions. In 35% (51/146) of these reviews they found discrepancies between what was planned in the protocol as registered in PROSPERO and what was reported on adverse effects in the final published reviews. Fourth, a wide body of evidence has shown that adverse events were often assessed inconsistently and reported inadequately in clinical trials and that most results on these events were not available in public sources [8, 40–42]. If these limitations also apply to the clinical trials that fed the reviews of this study one should further question the validity of the findings on adverse effect of systematic reviews of orthodontic interventions.

Strategies to improve the validity of what is reported on adverse effects of orthodontic interventions in systematic reviews include developing tailored core outcome sets on these effects [43] as well as guidelines for assessing and reporting them in both primary research and systematic reviews. Additional strategies on synthesizing adverse effects in systematic reviews at multiple levels were published in a recent paper by Qureshi et al. [19]. By implementing such strategies progress on the assessing and reporting of adverse effects of orthodontic interventions in both primary studies and systematic reviews can be made.

In conclusion the promising findings of this study should be interpreted with caution by its end users, because they could be jeopardized by numerous uncertainties. Much research is ahead to create valid and usable knowledge on adverse effects of orthodontic interventions involving a wide body of stakeholders.

## Supplementary Information

**Additional file 1.** STROBE Statement: Checklist of items that should be included in reports of cross-sectional studies [25].

**Additional file 2.** A. Differences between the protocol and the completed cross-sectional study. B. Selected journals and their 2018 impact factor (Clarivate Analytics 2021). C. Data collection forms*. D. Adverse effects hypothetically linked to orthodontic interventions according to Preoteasa et al. [24]*. E. Adverse effects hypothetically linked to orthodontic interventions*.

**Additional file 3.** Included reviews.

**Additional file 4.** Excluded studies.

## Data Availability

Not applicable.

## Abbreviations

AJODO: American Journal of Orthodontics and Dentofacial Orthopedics
AO: Angle Orthodontist
CONSORT: Consolidated Standards of Reporting Trials
EJO: European Journal of Orthodontics
KJO: Korean Journal of Orthodontics
O&CR: Orthodontics and Craniofacial Research
PRISMA: Preferred reporting items for systematic reviews and meta-analyses
PROSPERO: Prospective Register of Systematic Reviews
STROBE: Strengthening the Reporting of Observational Studies in Epidemiology
