# Supplementary material for: Seeking adverse effects in systematic reviews of orthodontic interventions: a cross-sectional study (part 1)
Source: Syst Rev. 2023 Jul 3;12:112. doi: 10.1186/s13643-023-02273-7 (PMC10318679; doi:10.1186/s13643-023-02273-7)
Supplement: Supplementary file 2 — Additional file 2. A. Differences between the protocol and the completed cross-sectional study. B. Selected journals and their 2018 impact factor (Clarivate Analytics 2021). C. Data collection forms*. D. Adverse effects hypothetically linked to orthodontic interventions according to Preoteasa et al. [24]*. E. Adverse effects hypothetically linked to orthodontic interventions*. [file 13643_2023_2273_MOESM2_ESM.docx]

**Additional file 2**

**Table of contents for additional file 2**

| **Page(s)** | **Additional file item** | **Description** |
| --- | --- | --- |
| 1 | Additional file 2A | Difference between the protocol and the completed cross-sectional study |
| 2 | Additional file 2B | Selected journals and their 2018 impact factor (Clarivate Analytics 2021) |
| 2-3 | Additional file 2C | Data collection forms |
| 4 | Additional file 2D | Adverse effects hypothetically linked to orthodontic interventions according to Preoteasa et al. (2012) |
| 5-6 | Additional file 2E | Adverse effects hypothetically linked to orthodontic interventions |
| 7 | References for additional file 2 | References for additional file 2 |

**Additional file 2A. Differences between the protocol and the completed cross-sectional study**

| **Differences between the protocol and the completed cross-sectional study** | **Rationale** |
| --- | --- |
| Extension of the search period to identify eligible systematic reviews | We planned to complete our study on July 31 2019, but as a result of COVID 19-related delays we extended our search period to July 31 2021. |
| Excluding systematic reviews with Bayesian network meta-analysis | We excluded systematic reviews with Bayesian network meta-analyses, because such reviews include the results of multiple interventions, which could make it difficult to understand which adverse effect was assigned to which specific intervention. |
| Excluding systematic reviews that did not assess a specific orthodontic intervention, but refer to orthodontic interventions as a whole. | We excluded systematic reviews that did not assess a specific orthodontic intervention, but referred to orthodontic interventions as a whole. |
| Excluding systematic reviews that were conducted by one operator only | We excluded reviews that were conducted by one reviewer only, because such an approach is often conducted for narrative reviews, but is not congruent with the systematic approach of systematic reviews. |
| Implementing univariable logistic regression models in the statistical analyses | We implemented univariable logistic regression models to determine the association between each of the 4 outcomes and the journal in which the SR was published using the Cochrane Database of Systematic Reviews as reference. Chi-square tests of independence as planned in our protocol were not used, because of the small number of systematic reviews in each eligible journal and the low variability in the response scored (prevalence of ‘no’ ranging from 9.2% to 15.3%). |
| In the protocol the following definition was used for orthodontic interventions: Steegmans et al. (2019a): ‘Orthodontic interventions refer to the use of any type of orthodontic appliance that are used to move teeth or change the jaw size or position for orthodontic purposes. These interventions also include appliances to maintain or stabilize the results of orthodontic treatment, for example retainers.’ | This definition was changed to: ‘Orthodontic interventions refer to the use of any type of orthodontic appliance to move teeth or change the jaw size or position for orthodontic purposes. These interventions also include appliances to maintain or stabilize the results of orthodontic treatment, for example retainers.’  Rationale for change: The modified definition is more accurate, but did not change the original meaning of the definition of orthodontic interventions |
| Inclusion of Dr. Nicola Di Girolama as an author | Dr. Di Girolamo was consulted for his assistance in statistical and methodological issues |

**Additional file 2B. Selected journals and their 2018 impact factor (Clarivate Analytics 2021)**

| **Journal** | **Impact factor** |
| --- | --- |
| Cochrane Database of Systematic reviews | 7.755 |
| American Journal of Orthodontics and Dentofacial Orthopedics | 1.911 |
| European Journal of Orthodontics | 1.841 |
| Korean Journal of Orthodontics | 1.476 |
| Orthodontics and Craniofacial Research | 0.946 |

**Additional file 2C. Data collection forms***

| **Items for the main manuscript** | **Description** |
| --- | --- |
| Journal | List the pertinent journal |
| Year | Year of publication |
| Binder page number | List the binder page number |
| Reference | List full reference (Authors, Title, Journal) |
| Is the article a systematic review? | Answer: Yes/No  Consider definition of a systematic review |
| Is the systematic review eligible? | Answer: Yes/No  Consult the eligibility criteria for addressing this answer. |
| What was the orthodontic intervention? | List the type of orthodontic intervention. |
| Was seeking of adverse effects of interventions defined as a research objective of the review? | Answer: Yes/No  Yes: When seeking of adverse effects of interventions was defined as a research objective or as a research question or when adverse effects were predefined a priori as outcomes to assess.  No: Seeking of adverse effects of interventions was not defined as a research objective or as a research question or when adverse effects were not predefined a priori as outcomes to assess. |
| What adverse effects of interventions were defined as research objectives? | Answer: List adverse effects/NA  List all adverse effects of interventions that the reviewers defined as research objectives.  NA: When the following question was answered with a ‘No’: ‘Was seeking of adverse effects of interventions defined as a research objective of the review?’ |
| Did the review seek any findings related to adverse effects of interventions in the included studies? | Answer: Yes/No  Yes: Any findings related to adverse effects of interventions in the included studies were sought by the reviewers.  Seeking any findings related to adverse effects of interventions in the included studies refers to reporting anywhere in the review (except in the Abstract) that such adverse effects in the included studies were sought.  Yes: Yes is also scored when reviewers only reported findings related to adverse effects of interventions in the included studies, but did not report that they actually sought them or planned to seek them. For example ‘Yes’ will be scored when outcomes on adverse effects of interventions in the included studies were reported in the review, but were not defined as objectives of the review.  Yes: Yes is also scored when the reviewers reported that they planned to seek (for example in the research objectives) findings related to adverse effects of interventions in the included studies, but did not report on these findings.  No: Findings related to adverse effects of interventions in the included studies were not sought by the reviewers. |
| Did the review report findings related to adverse effects of interventions sought in the included studies? | Answer: Yes/No  Yes: The review reported findings related to adverse effects of interventions sought in the included studies.  ‘Yes’ is also scored when the review reported that no findings on adverse effects of interventions in the included studies were identified.  No: The review did not report any findings related to adverse effects of interventions sought in the included studies. |
| What findings related to adverse effects of interventions sought in the included studies were reported in the review? | Answer: List of adverse effects  List all findings related to adverse effect(s) of interventions that were identified in the included studies and reported in the review. |
| Rationale for assigning an effect as 'adverse' or 'not adverse' (In case of additional or ambivalent adverse effects) | Answer: Present the rationale for assigning an effect as 'adverse' or 'not adverse' (In case of additional or ambivalent adverse effects) |
| Were potential adverse effects of the intervention considered, discussed (weighed) anywhere in the review? | Answer: Yes/No  Yes: Potential adverse effect(s) of interventions in the included studies were sought and reported by the reviewers. ‘Yes’ is also scored when potential adverse effect(s) of interventions were not sought, but only considered, discussed (weighed) anywhere (except in the Abstract) in the review.  ‘No’ is scored when potential adverse effects of the intervention were not considered, discussed (weighed) anywhere in the review. |
| Rationale for assigning an effect as 'adverse' or 'not adverse' (In case of additional or ambivalent adverse effects) | Answer: Present the rationale |

*To address our research question we will not consider what was reported regarding this question in the abstract and in the protocol of the review.

**Additional file 2D. Adverse effects hypothetically linked to orthodontic interventions according to Preoteasa et al. (2012)***

| **Local adverse effects** | |
| --- | --- |
| **Subgroup** | **Description** |
| Dental | - crown: decalcifications, decays, tooth wear, enamel cracks and fractures; discolorations, deterioration of prosthetic crown (as fracturing a ceramic one during debonding); - root: root resorption, early closure of root apex, ankylosis; - pulp: ischemia, pulpitis, necrosis; |
| Periodontal | - gingivitis, periodontitis, gingival recession or hypertrophy, alveolar bone loss, dehiscences, fenestrations, interdental fold, dark triangles; |
| Temporomandibular joint | - condylar resorption, temporomandibular dysfunction; |
| Soft tissues of the oral and maxillofacial region | - trauma (e.g., long archwires, headgear related), mucosal ulcerations or hyperplasia, chemical burns (e.g., etching related), thermal injuries (e.g., overheated burs), stomatitis, clumsy handling of dental instruments; |
| Unsatisfactory treatment outcome | - inadequate morpho-functional, aesthetic or functional final result, relapse, failure to complete treatment due to treatment dropout. |
| **Systemic adverse effects** | |
| **Subgroup** | **Description** |
| Psychological | - teasing, behavioral changes of patients and parents; discomfort associated with pain presence and aesthetic look discontents during orthodontic appliance usage; |
| Gastro-intestinal | - accidental swallowing of small parts of the orthodontic device (tubes, brackets); |
| Allergies | - to nickel or latex; |
| Cardiac | - infective endocarditis; |
| Chronic fatigue syndrome |  |
| Cross infections | - from doctor to patient, patient to doctor, patient to patient. |

*Permission to reproduce this table was obtained on August 16 2018 from InTech’s Publishing Ethics and Legal Affairs Department.

**Additional file 2E. Adverse effects hypothetically linked to orthodontic interventions***

| **Adverse effects related to** | **Description** |
| --- | --- |
| Tooth structures | Tooth crown   - decalcifications, decays, tooth wear, enamel cracks and fractures; discolorations, deterioration of prosthetic crown (as fracturing a ceramic one during debonding); - iatrogenic damage to the crown, e.g., fracture as a result of trauma   Tooth root   - root resorption, early closure of root apex, ankylosis; - iatrogenic damage to the root, e.g., fracture as a result of trauma   Tooth pulp   - ischemia, pulpitis, necrosis - iatrogenic damage to the pulp, e.g., fracture as a result of trauma |
| Periodontal tissues | - gingivitis, periodontitis, gingival recession or hypertrophy, alveolar bone loss, dehiscences, fenestrations, interdental fold, dark triangles; tooth mobility, plague retention, bacterial count |
| Intraoral (non-tooth or periodontal) tissues | - intraoral tissue irritations and inflammation such as mucosal ulcerations or hyperplasia or irritations of the tongue (as a result of trauma by appliances, e.g., breakage, failure, loosening etc. of appliances or long arch wires) - Scar formation after suturing - chemical burns (e.g., etching related) - thermal injuries (e.g., overheated burs) - nerve damage - tooth eruption, i.e., eruption disturbances (e.g., impactions) caused by orthodontic appliances |
| Extraoral tissues (non-temporomandibular tissues) | - cutting of lips or cheeks, eye injury (e.g., as a result of trauma by appliances, e.g., breakage, failure, loosening etc. of appliances or long arch wires or headgear-related trauma) - discomfort on the lip |
| Temporomandibular tissues and disorders | - temporomandibular tissues and disorders |
| Appliance failure | - breakage, failure, loosening etc. of appliances - long archwires, headgear-related trauma |
| Undesired treatment results | - inadequate morpho-functional, aesthetic or functional final result - inaccuracy of the treatment result - non predictability of the treatment result - Dental side effects e.g., unwanted tipping of teeth, anchorage loss etc. - Skeletal side effects, e.g., unwanted backward rotation of the mandible |
| Relapse and stability | - Relapse and stability of the obtained treatment result |
| Undesired qualitative experiences by the patient or carer(s) | Pain and discomfort   - orthodontic tooth movement-related pain and discomfort - appliance (intervention)-related pain and discomfort: i.e., pain and discomfort as a result of the appliance (intervention) itself with or without pain and discomfort associated with tooth movement e.g., tension or pressure of the appliances (constriction of appliances), speech difficulties, eating difficulties, swallowing difficulties, food accumulation, bad tastes and smells - additional intervention-related pain and discomfort, e.g., surgical and non-surgical adjunctive interventions to accelerate tooth movement   Tolerability/acceptance/stress issues with the treatment procedures   - Absence from work or studies and difficulties in daily activities - collaboration (compliance) issues or failure to complete treatment, e.g., dropout - patient anxiety - being teased - social discomfort - embarrassment to wear the appliance - behavioral changes of patients and parents, impaired family relationships - aesthetic look discontents during orthodontic appliance usage - concentration difficulties - reduced enjoyment of food and change in taste - sleeping difficulties - removal of appliance during sleep - development of mannerisms   Satisfaction with the treatment procedures and final result   - not satisfied with the treatment procedures (Check in text what was measured, i.e., during or after) - not satisfied with the final treatment result (Check in text what was measured, i.e., during or after) |
| Gastro-intestinal | - accidental swallowing parts of the orthodontic device (tubes, brackets); |
| Allergy | - Allergies to nickel or latex; |
| Cardio | - infective endocarditis; |
| Chronic fatigue |  |
| Cross infections | - from doctor to patient, patient to doctor, patient to patient. |
| Non-defined | Adverse effects that were not defined by the authors of the review: referring to ‘any adverse effect’, ‘any side effect’ etc. |
| Additional adverse effects | Additional adverse effects that were identified during data extraction that could not be labeled under any of the categories of adverse effects given in this table |

*Modified from Preoteasa et al. (Preoteasa 2012)

**References for additional file 2**

**Clarivate Analytics 2021**

Clarivate Analytics. [online] Available from: <https://clarivate.com/> (accessed December 4^th^ 2021).

**Preoteasa 2012**

Preoteasa CT, Ionescu E, Preoteasa E. Chapter 18: Risks and complications associated with orthodontic treatment. In: Bourzgui F. (editor). Orthodontics-Basic aspects and clinical considerations. March 9, 2012 under CC BY 3.0 license. www.intechopen.com. [online] Available from:

<https://cdn.intechopen.com/pdfs/31388/InTech-Risks_and_complicationsassociated_with_orthodontic_treatment.pdf> (accessed December 4^th^ 2021).

**Steegmans 2019a**

Steegmans PAJ, Bipat S, Meursinge Reynders RA. Seeking adverse effects in systematic reviews of orthodontic interventions: protocol for a cross-sectional study. Syst Rev. 2019 Apr 5;8(1):89. doi: 10.1186/s13643-019-1000-1. PMID: 30953538; PMCID: PMC6449933.
