# Supplementary material for: Seeking adverse effects in systematic reviews of orthodontic interventions: a cross-sectional study (part 1)
Source: Syst Rev. 2023 Jul 3;12:112. doi: 10.1186/s13643-023-02273-7 (PMC10318679; doi:10.1186/s13643-023-02273-7)
Supplement: Supplementary file 3 — Additional file 3. Included reviews. [file 13643_2023_2273_MOESM3_ESM.docx]

**Additional file 3. Included reviews**

**Included systematic reviews of orthodontic interventions**

| **Journal^*^** | **Year** | **Reference** |
| --- | --- | --- |
| Cochrane library | 2018 | Batista KB, Thiruvenkatachari B, Harrison JE, O'Brien KD. Orthodontic treatment for prominent upper front teeth (Class II malocclusion) in children and adolescents.Cochrane Database Syst Rev. 2018 Mar 13;3:CD003452. doi: 10.1002/14651858.CD003452.pub4. |
| Cochrane library | 2018 | Wang Y, Liu C, Jian F, McIntyre GT, Millett DT, Hickman J, Lai W. Initial arch wires used in orthodontic treatment with fixed appliances.Cochrane Database Syst Rev. 2018 Jul 31;7:CD007859. doi: 10.1002/14651858.CD007859.pub4. |
| Cochrane library | 2015 | Borrie FR, Bearn DR, Innes NP, Iheozor-Ejiofor Z. Interventions for the cessation of non-nutritive sucking habits in children. Cochrane Database Syst Rev. 2015 Mar 31;(3):CD008694. doi: 10.1002/14651858.CD008694.pub2. |
| Cochrane library | 2015 | Fleming PS, Fedorowicz Z, Johal A, El-Angbawi A, Pandis N. Surgical adjunctive procedures for accelerating orthodontic treatment. Cochrane Database Syst Rev. 2015 Jun 30;(6):CD010572. doi: 10.1002/14651858.CD010572.pub2. |
| Cochrane library | 2015 | El-Angbawi A, McIntyre GT, Fleming PS, Bearn DR. Non-surgical adjunctive interventions for accelerating tooth movement in patients undergoing fixed orthodontic treatment. Cochrane Database Syst Rev. 2015 Nov 18;(11):CD010887. doi: 10.1002/14651858.CD010887.pub2. |
| Cochrane library | 2014 | Agostino P, Ugolini A, Signori A, Silvestrini-Biavati A, Harrison JE, Riley P. Orthodontic treatment for posterior crossbites. Cochrane Database Syst Rev. 2014 Aug 8;(8):CD000979. doi: 10.1002/14651858.CD000979.pub2. |
| Cochrane library | 2014 | Jambi S, Walsh T, Sandler J, Benson PE, Skeggs RM, O'Brien KD. Reinforcement of anchorage during orthodontic brace treatment with implants or other surgical methods. Cochrane Database Syst Rev. 2014 Aug 19;(8):CD005098. doi: 10.1002/14651858.CD005098.pub3. |
| Cochrane library | 2014 | Lentini-Oliveira DA, Carvalho FR, Rodrigues CG, Ye Q, Prado LB, Prado GF, Hu R. Orthodontic and orthopaedic treatment for anterior open bite in children. Cochrane Database Syst Rev. 2014 Sep 24;(9):CD005515. doi: 10.1002/14651858.CD005515.pub3. |
| Cochrane library | 2013 | Watkinson S, Harrison JE, Furness S, Worthington HV.Orthodontic treatment for prominent lower front teeth (Class III malocclusion) in children. Cochrane Database Syst Rev. 2013 Sep 30;(9):CD003451. doi: 10.1002/14651858.CD003451.pub2. |
| Cochrane library | 2013 | Jambi S, Thiruvenkatachari B, O'Brien KD, Walsh T. Orthodontic treatment for distalising upper first molars in children and adolescents. Cochrane Database Syst Rev. 2013 Oct 23;(10):CD008375. doi: 10.1002/14651858.CD008375.pub2. |
| EJO | 2021 | Afzal E, Fida M, Malik DS, Irfan S, Gul M. Comparison between conventional and piezocision-assisted orthodontics in relieving anterior crowding: a systematic review and meta-analysis. Eur J Orthod. 2021 Jun 8;43(3):360-366. doi: 10.1093/ejo/cjaa046. PMID: 32812636. |
| EJO | 2021 | Kapetanović A, Theodorou CI, Bergé SJ, Schols JGJH, Xi T. Efficacy of Miniscrew-Assisted Rapid Palatal Expansion (MARPE) in late adolescents and adults: a systematic review and meta-analysis. Eur J Orthod. 2021 Jun 8;43(3):313-323. doi: 10.1093/ejo/cjab005. PMID: 33882127; PMCID: PMC8186837. |
| EJO | 2021 | Rutili V, Mrakic G, Nieri M, Franceschi D, Pierleoni F, Giuntini V, Franchi L. Dento-skeletal effects produced by rapid versus slow maxillary expansion using fixed jackscrew expanders: a systematic review and meta-analysis. Eur J Orthod. 2021 Jun 8;43(3):301-312. doi: 10.1093/ejo/cjaa086. PMID: 33950178. |
| EJO | 2021 | Cornelis MA, Tepedino M, Riis NV, Niu X, Cattaneo PM. Treatment effect of bone-anchored maxillary protraction in growing patients compared to controls: a systematic review with meta-analysis. Eur J Orthod. 2021 Jan 29;43(1):51-68. doi: 10.1093/ejo/cjaa016. PMID: 32815989. |
| EJO | 2020 | Shahabee M, Shafaee H, Abtahi M, Rangrazi A, Bardideh E. Effect of micro-osteoperforation on the rate of orthodontic tooth movement-a systematic review and a meta-analysis. Eur J Orthod. 2020 Apr 1;42(2):211-221. doi: 10.1093/ejo/cjz049. PMID: 31215993. |
| EJO | 2020 | González Espinosa D, Santos M, Mendes SMDA, Normando D. Mandibular propulsion appliance for adults with Class II malocclusion: a systematic review and meta-analysis. Eur J Orthod. 2020 Apr 1;42(2):163-173. doi: 10.1093/ejo/cjz089. PMID: 31786599. |
| EJO | 2020 | Mohammed H, Čirgić E, Rizk MZ, Vandevska-Radunovic V. Effectiveness of prefabricated myofunctional appliances in the treatment of Class II division 1 malocclusion: a systematic review. Eur J Orthod. 2020 Apr 1;42(2):125-134. doi: 10.1093/ejo/cjz025. PMID: 31329848. |
| EJO | 2019 | Lyu C, Zhang L, Zou S. The effectiveness of supplemental vibrational force on enhancing orthodontic treatment. A systematic review. Eur J Orthod. 2019 Sep 21;41(5):502-512. doi: 10.1093/ejo/cjz018. PMID: 31065683. |
| EJO | 2018 | Algharbi M, Bazargani F, Dimberg L. Do different maxillary expansion appliances influence the outcomes of the treatment?Eur J Orthod. 2018 Jan 23;40(1):97-106. doi: 10.1093/ejo/cjx035. |
| EJO | 2018 | Al Rahma WJ, Kaklamanos EG, Athanasiou AE. Performance of Hawley-type retainers: a systematic review of randomized clinical trials.Eur J Orthod. 2018 Apr 6;40(2):115-125. doi: 10.1093/ejo/cjx036. |
| EJO | 2017 | Feres MF, Abreu LG, Insabralde NM, de Almeida MR, Flores-Mir . Effectiveness of open bite correction when managing deleterious oral habits in growing children and adolescents: a systematic review and meta-analysis. Eur J Orthod. 2017 Feb;39(1):31-42. doi: 10.1093/ejo/cjw005. Epub 2016 Feb 3. |
| EJO | 2017 | Papageorgiou SN, Kutschera E, Memmert S, Gölz L, Jäger A, Bourauel C, Eliades T. Effectiveness of early orthopaedic treatment with headgear: a systematic review and meta-analysis. Eur J Orthod. 2017 Apr 1;39(2):176-187. doi: 10.1093/ejo/cjw041. |
| EJO | 2016 | Zymperdikas VF, Koretsi V, Papageorgiou SN, Papadopoulos MA. Treatment effects of fixed functional appliances in patients with Class II malocclusion: a systematic review and meta-analysis. Eur J Orthod. 2016 Apr;38(2):113-26. doi: 10.1093/ejo/cjv034. |
| EJO | 2016 | Feres MF, Abreu LG, Insabralde NM, Almeida MR, Flores-Mir C. Effectiveness of the open bite treatment in growing children and adolescents. A systematic review. Eur J Orthod. 2016 Jun;38(3):237-50. doi: 10.1093/ejo/cjv048. |
| EJO | 2016 | Yang X, Zhu Y, Long H, Zhou Y, Jian F, Ye N, Gao M, Lai W. The effectiveness of the Herbst appliance for patients with Class II malocclusion: a meta-analysis. Eur J Orthod. 2016 Jun;38(3):324-33. doi: 10.1093/ejo/cjv057. |
| EJO | 2016 | Mistakidis I, Katib H, Vasilakos G, Kloukos D, Gkantidis N. Clinical outcomes of lingual orthodontic treatment: a systematic review. Eur J Orthod. 2016 Oct;38(5):447-58. doi: 10.1093/ejo/cjv061. |
| EJO | 2016 | Elkordy SA, Aboelnaga AA, Fayed MM, AboulFotouh MH, Abouelezz AM.Can the use of skeletal anchors in conjunction with fixed functional appliances promote skeletal changes? A systematic review and meta-analysis. Eur J Orthod. 2016 Oct;38(5):532-45. doi: 10.1093/ejo/cjv081. |
| EJO | 2016 | Pacha MM, Fleming PS, Johal A. A comparison of the efficacy of fixed versus removable functional appliances in children with Class II malocclusion: A systematic review. Eur J Orthod. 2016 Dec;38(6):621-630. |
| EJO | 2015 | Ehsani S, Nebbe B, Normando D, Lagravere MO, Flores-Mir C. Short-term treatment effects produced by the Twin-block appliance: a systematic review and meta-analysis. Eur J Orthod. 2015 Apr;37(2):170-6. doi: 10.1093/ejo/cju030. |
| EJO | 2015 | Zurfluh MA, Kloukos D, Patcas R, Eliades T. Effect of chin-cup treatment on the temporomandibular joint: a systematic review. Eur J Orthod. 2015 Jun;37(3):314-24. doi: 10.1093/ejo/cju048. |
| EJO | 2015 | Koretsi V, Zymperdikas VF, Papageorgiou SN, Papadopoulos MA. Treatment effects of removable functional appliances in patients with Class II malocclusion: a systematic review and meta-analysis. Eur J Orthod. 2015 Aug;37(4):418-34. doi: 10.1093/ejo/cju071. |
| EJO | 2015 | Liu S, Xu T, Zou W. Effects of rapid maxillary expansion on the midpalatal suture: a systematic review. Eur J Orthod. 2015 Dec;37(6):651-5. doi: 10.1093/ejo/cju100. |
| EJO | 2014 | Zhou Y, Long H, Ye N, Xue J, Yang X, Liao L, Lai W. The effectiveness of non-surgical maxillary expansion: a meta-analysis. Eur J Orthod. 2014 Apr;36(2):233-42. doi: 10.1093/ejo/cjt044. |
| EJO | 2014 | Papageorgiou SN, Konstantinidis I, Papadopoulou K, Jäger A, Bourauel C. Clinical effects of pre-adjusted edgewise orthodontic brackets: a systematic review and meta-analysis. Eur J Orthod. 2014 Jun;36(3):350-63. doi: 10.1093/ejo/cjt064. |
| EJO | 2013 | Zuccati G, Casci S, Doldo T, Clauser C. Expansion of maxillary arches with crossbite: a systematic review of RCTs in the last 12 years. Eur J Orthod. 2013 Feb;35(1):29-37. doi: 10.1093/ejo/cjr140. |
| EJO | 2013 | Fleming PS, Johal A, Pandis N. The effectiveness of laceback ligatures during initial orthodontic alignment: a systematic review and meta-analysis. Eur J Orthod. 2013 Aug;35(4):539-46. doi: 10.1093/ejo/cjs033. |
| EJO | 2011 | Perillo L, Cannavale R, Ferro F, Franchi L, Masucci C, Chiodini P, Baccetti T. Meta-analysis of skeletal mandibular changes during Frankel appliance treatment. Eur J Orthod. 2011 Feb;33(1):84-92. doi: 10.1093/ejo/cjq033. |
| EJO | 2011 | Naoumova J, Kurol J, Kjellberg H. A systematic review of the interceptive treatment of palatally displaced maxillary canines. Eur J Orthod. 2011 Apr;33(2):143-9. doi: 10.1093/ejo/cjq045. |
| AJODO | 2020 | Santana LG, de Campos França E, Flores-Mir C, Abreu LG, Marques LS, Martins-Junior PA. Effects of lip bumper therapy on the mandibular arch dimensions of children and adolescents: A systematic review. Am J Orthod Dentofacial Orthop. 2020 Apr;157(4):454-465.e1. doi: 10.1016/j.ajodo.2019.10.014. PMID: 32241352. |
| AJODO | 2020 | Sivarajan S, Ringgingon LP, Fayed MMS, Wey MC. The effect of micro-osteoperforations on the rate of orthodontic tooth movement: A systematic review and meta-analysis. Am J Orthod Dentofacial Orthop. 2020 Mar;157(3):290-304. doi: 10.1016/j.ajodo.2019.10.009. PMID: 32115107. |
| AJODO | 2019 | Theodorou CI, Kuijpers-Jagtman AM, Bronkhorst EM, Wagener FADTG. Optimal force magnitude for bodily orthodontic tooth movement with fixed appliances: A systematic review. Am J Orthod Dentofacial Orthop. 2019 Nov;156(5):582-592. doi: 10.1016/j.ajodo.2019.05.011. PMID: 31677666. |
| AJODO | 2018 | Kouvelis G, Dritsas K, Doulis I, Kloukos D, Gkantidis N. Effect of orthodontic treatment with 4 premolar extractions compared with nonextraction treatment on the vertical dimension of the face: A systematic review.Am J Orthod Dentofacial Orthop. 2018 Aug;154(2):175-187. doi: 10.1016/j.ajodo.2018.03.007. |
| AJODO | 2018 | Aljabaa A, Almoammar K, Aldrees A, Huang G. Effects of vibrational devices on orthodontic tooth movement: A systematic review.Am J Orthod Dentofacial Orthop. 2018 Dec;154(6):768-779. doi: 10.1016/j.ajodo.2018.07.012. |
| AJODO | 2017 | Woon SC, Thiruvenkatachari B. Early orthodontic treatment for Class III malocclusion: A systematic review and meta-analysis. Am J Orthod Dentofacial Orthop. 2017 Jan;151(1):28-52. doi: 10.1016/j.ajodo.2016.07.017. |
| AJODO | 2017 | Antoszewska-Smith J, Sarul M, Łyczek J, Konopka T, Kawala B. Effectiveness of orthodontic miniscrew implants in anchorage reinforcement during en-masse retraction: A systematic review and meta-analysis. Am J Orthod Dentofacial Orthop. 2017 Mar;151(3):440-455. doi: 10.1016/j.ajodo.2016.08.029. |
| AJODO | 2016 | Nucera R, Lo Giudice A, Rustico L, Matarese G, Papadopoulos MA, Cordasco G. Effectiveness of orthodontic treatment with functional appliances on maxillary growth in the short term: A systematic review and meta-analysis. Am J Orthod Dentofacial Orthop. 2016 May;149(5):600-611.e3. doi: 10.1016/j.ajodo.2015.09.030. |
| AJODO | 2016 | Ishaq RA, AlHammadi MS, Fayed MM, El-Ezz AA, Mostafa Y. Fixed functional appliances with multibracket appliances have no skeletal effect on the mandible: A systematic review and meta-analysis. Am J Orthod Dentofacial Orthop. 2016 May;149(5):612-24. doi: 10.1016/j.ajodo.2015.11.023. |
| AJODO | 2016 | Silveira GS, de Almeida NV, Pereira DM, Mattos CT, Mucha JN. Prosthetic replacement vs space closure for maxillary lateral incisor agenesis: A systematic review. Am J Orthod Dentofacial Orthop. 2016 Aug;150(2):228-37. doi: 10.1016/j.ajodo.2016.01.018. |
| AJODO | 2015 | Thiruvenkatachari B, Harrison J, Worthington H, O'Brien K. Early orthodontic treatment for Class II malocclusion reduces the chance of incisal trauma: Results of a Cochrane systematic review. Am J Orthod Dentofacial Orthop. 2015 Jul;148(1):47-59. doi: 10.1016/j.ajodo.2015.01.030. |
| AJODO | 2014 | Hoogeveen EJ, Jansma J, Ren Y. Surgically facilitated orthodontic treatment: a systematic review. Am J Orthod Dentofacial Orthop. 2014 Apr;145(4 Suppl):S51-64. doi: 10.1016/j.ajodo.2013.11.019. |
| AJODO | 2014 | Yang X, Li C, Bai D, Su N, Chen T, Xu Y, Han X. Treatment effectiveness of Fränkel function regulator on the Class III malocclusion: a systematic review and meta-analysis. Am J Orthod Dentofacial Orthop. 2014 Aug;146(2):143-54. doi: 10.1016/j.ajodo.2014.04.017. |
| AJODO | 2013 | Janson G, Sathler R, Fernandes TM, Branco NC, Freitas MR. Correction of Class II malocclusion with Class II elastics: a systematic review.Am J Orthod Dentofacial Orthop. 2013 Mar;143(3):383-92. doi: 10.1016/j.ajodo.2012.10.015. |
| AJODO | 2013 | Grec RH, Janson G, Branco NC, Moura-Grec PG, Patel MP, Castanha Henriques JF. Intraoral distalizer effects with conventional and skeletal anchorage: a meta-analysis. Am J Orthod Dentofacial Orthop. 2013 May;143(5):602-15. doi: 10.1016/j.ajodo.2012.11.024. |
| AJODO | 2012 | Millett DT, Cunningham SJ, O'Brien KD, Benson PE, de Oliveira CM. Treatment and stability of class II division 2 malocclusion in children and adolescents: a systematic review. Am J Orthod Dentofacial Orthop. 2012 Aug;142(2):159-169.e9. doi: 10.1016/j.ajodo.2012.03.022. |
| AJODO | 2011 | Marsico E, Gatto E, Burrascano M, Matarese G, Cordasco G. Effectiveness of orthodontic treatment with functional appliances on mandibular growth in the short term. Am J Orthod Dentofacial Orthop. 2011 Jan;139(1):24-36. doi: 10.1016/j.ajodo.2010.04.028. |
| AJODO | 2011 | Fudalej P, Antoszewska J. Are orthodontic distalizers reinforced with the temporary skeletal anchorage devices effective? Am J Orthod Dentofacial Orthop. 2011 Jun;139(6):722-9. doi: 10.1016/j.ajodo.2011.01.019. |
| AJODO | 2011 | Baratieri C, Alves M Jr, de Souza MM, de Souza Araújo MT, Maia LC. Does rapid maxillary expansion have long-term effects on airway dimensions and breathing? Am J Orthod Dentofacial Orthop. 2011 Aug;140(2):146-56. doi: 10.1016/j.ajodo.2011.02.019. |
| AJODO | 2010 | Chen SS, Greenlee GM, Kim JE, Smith CL, Huang GJ. Systematic review of self-ligating brackets. Am J Orthod Dentofacial Orthop. 2010 Jun;137(6):726.e1-726.e18; discussion 726-7. doi: 10.1016/j.ajodo.2009.11.009. |
| AO | 2021 | Liu L, Zhan Q, Zhou J, Kuang Q, Yan X, Zhang X, Shan Y, Lai W, Long H. A comparison of the effects of Forsus appliances with and without temporary anchorage devices for skeletal Class II malocclusion. Angle Orthod. 2021 Mar 1;91(2):255-266. doi: 10.2319/051120-421.1. PMID: 33378419; PMCID: PMC8028478. |
| AO | 2020 | Torres D, Lopes J, Magno MB, Cople Maia L, Normando D, Leão PB. Effects of rapid maxillary expansion on temporomandibular joints. Angle Orthod. 2020 May 1;90(3):442-456. doi: 10.2319/080619-517.1. PMID: 33378434; PMCID: PMC8032308. |
| AO | 2020 | Sosly R, Mohammed H, Rizk MZ, Jamous E, Qaisi AG, Bearn DR. Effectiveness of miniscrew-supported maxillary incisor intrusion in deep-bite correction: *A systematic review and meta-analysis*. Angle Orthod. 2020 Mar;90(2):291-304. doi: 10.2319/061119-400.1. Epub 2019 Dec 9. PMID: 31816252; PMCID: PMC8051239. |
| AO | 2018 | Vieira EP, Watanabe BSD, Pontes LF, Mattos JNF, Maia LC, Normando D. The effect of bracket slot size on the effectiveness of orthodontic treatment: A systematic review.Angle Orthod. 2018 Jan;88(1):100-106. doi: 10.2319/031217-185.1. |
| AO | 2018 | Mohamed RN, Basha S, Al-Thomali Y. Maxillary molar distalization with miniscrew-supported appliances in Class II malocclusion: A systematic review.Angle Orthod. 2018 Jul;88(4):494-502. doi: 10.2319/091717-624.1. |
| AO | 2018 | Ellabban MT, Abdul-Aziz AI, Fayed MMS, AboulFotouh MH, Elkattan ES, Dahaba MM. Positional and dimensional temporomandibular joint changes after correction of posterior crossbite in growing patients: A systematic review.Angle Orthod. 2018 Sep;88(5):638-648. doi: 10.2319/110217-749.1. |
| AO | 2017 | Xu Y, Xie J. Comparison of the effects of mini-implant and traditional anchorage on patients with maxillary dentoalveolar protrusion. Angle Orthod. 2017 Mar;87(2):320-327. doi: 10.2319/051016-375.1. |
| AO | 2017 | Janson G, Aliaga-Del Castillo A, Niederberger A. Changes in apical base sagittal relationship in Class II malocclusion treatment with and without premolar extractions: A systematic review and meta-analysis. Angle Orthod. 2017 Mar;87(2):338-355. doi: 10.2319/030716-198.1. |
| AO | 2017 | Yi J, Xiao J, Li Y, Li X, Zhao Z. Efficacy of piezocision on accelerating orthodontic tooth movement: A systematic review. Angle Orthod. 2017 Jul;87(4):491-498. doi: 10.2319/01191-751.1. |
| AO | 2017 | Fagundes NCF, Rabello NM, Maia LC, Normando D, Mello KCFR. Can rapid maxillary expansion cause auditory improvement in children and adolescents with hearing loss? A systematic review. Angle Orthod. 2017 Nov;87(6):886-896. doi: 10.2319/021517-111.1. |
| AO | 2015 | Perinetti G, Primožič J, Furlani G, Franchi L, Contardo L. Treatment effects of fixed functional appliances alone or in combination with multibracket appliances: A systematic review and meta-analysis. Angle Orthod. 2015 May;85(3):480-92. doi: 10.2319/102813-790.1. |
| AO | 2015 | Lopes Filho H, Maia LH, Lau TC, de Souza MM, Maia LC. Early vs late orthodontic treatment of tooth crowding by first premolar extraction: A systematic review. Angle Orthod. 2015 May;85(3):510-7. doi: 10.2319/050814-332.1. |
| AO | 2015 | Rossini G, Parrini S, Castroflorio T, Deregibus A, Debernardi CL. Efficacy of clear aligners in controlling orthodontic tooth movement: a systematic review.Angle Orthod. 2015 Sep;85(5):881-9. doi: 10.2319/061614-436.1. |
| AO | 2015 | Feres MF, Raza H, Alhadlaq A, El-Bialy T. Rapid maxillary expansion effects in Class II malocclusion: a systematic review.Angle Orthod. 2015 Nov;85(6):1070-9. doi: 10.2319/102514-768.1. |
| AO | 2013 | Long H, Pyakurel U, Wang Y, Liao L, Zhou Y, Lai W. Interventions for accelerating orthodontic tooth movement: a systematic review. Angle Orthod. 2013 Jan;83(1):164-71. doi: 10.2319/031512-224.1. |
| AO | 2013 | Flores-Mir C, McGrath L, Heo G, Major PW. Efficiency of molar distalization associated with second and third molar eruption stage. Angle Orthod. 2013 Jul;83(4):735-42. doi: 10.2319/081612-658.1. |
| AO | 2013 | Bazargani F, Feldmann I, Bondemark L. Three-dimensional analysis of effects of rapid maxillary expansion on facial sutures and bones. Angle Orthod. 2013 Nov;83(6):1074-82. doi: 10.2319/020413-103.1. |
| AO | 2012 | Feng X, Li J, Li Y, Zhao Z, Zhao S, Wang J. Effectiveness of TAD-anchored maxillary protraction in late mixed dentition. Angle Orthod. 2012 Nov;82(6):1107-14. doi: 10.2319/111411-705.1. |
| AO | 2011 | Liu ZP, Li CJ, Hu HK, Chen JW, Li F, Zou SJ. Efficacy of short-term chincup therapy for mandibular growth retardation in Class III malocclusion. Angle Orthod. 2011 Jan;81(1):162-68. doi: 10.2319/050510-244.1. |
| AO | 2011 | Li F, Hu HK, Chen JW, Liu ZP, Li GF, He SS, Zou SJ, Ye QS. Comparison of anchorage capacity between implant and headgear during anterior segment retraction. Angle Orthod. 2011 Sep;81(5):915-22. doi: 10.2319/101410-603.1. |
| AO | 2010 | Leonardi R, Annunziata A, Licciardello V, Barbato E. Soft tissue changes following the extraction of premolars in nongrowing patients with bimaxillary protrusion. A systematic review. Angle Orthod. 2010 Jan;80(1):211-6. doi: 10.2319/010709-16.1. |
| AO | 2010 | Fleming PS, Johal A. Self-ligating brackets in orthodontics. A systematic review. Angle Orthod. 2010 May;80(3):575-84. doi: 10.2319/081009-454.1. |
| AO | 2009 | Gordon JM, Rosenblatt M, Witmans M, Carey JP, Heo G, Major PW, Flores-Mir C. Rapid palatal expansion effects on nasal airway dimensions as measured by acoustic rhinometry. A systematic review. Angle Orthod. 2009 Sep;79(5):1000-7. doi: 10.2319/082108-441.1. |
| KJO | 2020 | Giudice AL, Spinuzza P, Rustico L, Messina G, Nucera R. Short-term treatment effects produced by rapid maxillary expansion evaluated with computed tomography: A systematic review with meta-analysis. Korean J Orthod. 2020 Sep 25;50(5):314-323. doi: 10.4041/kjod.2020.50.5.314. PMID: 32938824; PMCID: PMC7500570. |
| KJO | 2019 | Galan-Lopez L, Barcia-Gonzalez J, Plasencia E. A systematic review of the accuracy and efficiency of dental movements with Invisalign. Korean J Orthod. 2019 May;49(3):140-149. doi: 10.4041/kjod.2019.49.3.140. Epub 2019 May 21. |
| KJO | 2018 | Viwattanatipa N, Charnchairerk S. The effectiveness of corticotomy and piezocision on canine retraction: A systematic review.Korean J Orthod. 2018 May;48(3):200-211. doi: 10.4041/kjod.2018.48.3.200. |
| O&CR | 2021 | Bayome M, Park JH, Bay C, Kook YA. Distalization of maxillary molars using temporary skeletal anchorage devices: A systematic review and meta-analysis. Orthod Craniofac Res. 2021 Mar;24 Suppl 1:103-112. doi: 10.1111/ocr.12470. Epub 2021 Feb 8. |
| O&CR | 2021 | Arvind P TR, Jain RK. Skeletally anchored forsus fatigue resistant device for correction of Class II malocclusions-A systematic review and meta-analysis. Orthod Craniofac Res. 2021 Feb;24(1):52-61. doi: 10.1111/ocr.12414. Epub 2020 Sep 7. PMID: 32772479. |
| O&CR | 2021 | MacDonald, L., Zanjir, M., Laghapour Lighvan, N., da Costa, B. R., Suri, S., & Azarpazhooh, A. (2020). Efficacy and Safety of Different Interventions to Accelerate Maxillary Canine Retraction Following Premolar Extraction: A Systematic Review and Network Meta‐analysis. Orthodontics & Craniofacial Research. doi:10.1111/ocr.12409 |
| O&CR | 2020 | Santana LG, Avelar K, Flores-Mir C, Marques LS. Incremental or maximal mandibular advancement in the treatment of class II malocclusion through functional appliances: A systematic review with meta-analysis. Orthod Craniofac Res. 2020 Nov;23(4):371-384. doi: 10.1111/ocr.12388. Epub 2020 May 29. PMID: 32390332. |
| O&CR | 2020 | Zhang B, Huang X, Huo S, Zhang C, Zhao S, Cen X, Zhao Z. Effect of clear aligners on oral health-related quality of life: A systematic review. Orthod Craniofac Res. 2020 Nov;23(4):363-370. doi: 10.1111/ocr.12382. Epub 2020 May 13. PMID: 32340082. |
| O&CR | 2020 | Niu X, Di Carlo G, Cornelis MA, Cattaneo PM. Three-dimensional analyses of short- and long-term effects of rapid maxillary expansion on nasal cavity and upper airway: A systematic review and meta-analysis. Orthod Craniofac Res. 2020 Aug;23(3):250-276. doi: 10.1111/ocr.12378. Epub 2020 May 5. PMID: 32248642. |
| O&CR | 2020 | Robertson L, Kaur H, Fagundes NCF, Romanyk D, Major P, Flores Mir C. Effectiveness of clear aligner therapy for orthodontic treatment: A systematic review. Orthod Craniofac Res. 2020 May;23(2):133-142. doi: 10.1111/ocr.12353. Epub 2019 Nov 13. PMID: 31651082. |
| O&CR | 2019 | Dab S, Chen K, Flores-Mir C. Short- and long-term potential effects of accelerated osteogenic orthodontic treatment: A systematic review and meta-analysis. Orthod Craniofac Res. 2019 May;22(2):61-68. doi: 10.1111/ocr.12272. Epub 2019 Mar 18. |
| O&CR | 2018 | Lee WC, Tu YK2, Huang CS, Chen R, Fu MW, Fu E. Pharyngeal airway changes following maxillary expansion or protraction: A meta-analysis.Orthod Craniofac Res. 2018 Feb;21(1):4-11. doi: 10.1111/ocr.12208. |
| O&CR | 2018 | Mohammed H, Rizk MZ, Wafaie K, Almuzian M. Effectiveness of nickel-titanium springs vs elastomeric chains in orthodontic space closure: A systematic review and meta-analysis.Orthod Craniofac Res. 2018 Feb;21(1):12-19. doi: 10.1111/ocr.12210. |
| O&CR | 2018 | Koletsi D, Makou M, Pandis N. Effect of orthodontic management and orofacial muscle training protocols on the correction of myofunctional and myoskeletal problems in developing dentition. A systematic review and meta-analysis.Orthod Craniofac Res. 2018 Nov;21(4):202-215. doi: 10.1111/ocr.12240. |
| O&CR | 2017 | Zheng M, Liu R, Ni Z, Yu Z. Efficiency, effectiveness and treatment stability of clear aligners: A systematic review and meta-analysis. Orthod Craniofac Res. 2017 Aug;20(3):127-133. doi: 10.1111/ocr.12177. |
| O&CR | 2014 | Cordasco G, Matarese G, Rustico L, Fastuca S, Caprioglio A, Lindauer SJ, Nucera R. Efficacy of orthopedic treatment with protraction facemask on skeletal Class III malocclusion: a systematic review and meta-analysis. Orthod Craniofac Res. 2014 Aug;17(3):133-43. doi: 10.1111/ocr.12040. |
| O&CR | 2014 | Papageorgiou SN, Konstantinidis I, Papadopoulou K, Jäger A, Bourauel C. A systematic review and meta-analysis of experimental clinical evidence on initial aligning archwires and archwire sequences. Orthod Craniofac Res. 2014 Nov;17(4):197-215. doi: 10.1111/ocr.12048. |

*Description of the abbreviated journals:

Cochrane library: Cochrane Database of Systematic Reviews

AJODO: American Journal of Orthodontics and Dentofacial Orthopedics

EJO: European Journal of Orthodontics

AO: Angle Orthodontist

KJO: Korean Journal of Orthodontics

O&CR: Orthodontics and Craniofacial Research
