# Supplementary material for: Seeking adverse effects in systematic reviews of orthodontic interventions: a cross-sectional study (part 1)
Source: Syst Rev. 2023 Jul 3;12:112. doi: 10.1186/s13643-023-02273-7 (PMC10318679; doi:10.1186/s13643-023-02273-7)
Supplement: Supplementary file 4 — Additional file 4. Excluded studies. [file 13643_2023_2273_MOESM4_ESM.docx]

**Additional file 4. Excluded studies**

**Update**

The following reference from 2017: Arora A, Khattri S, Ismail NM, Kumbargere Nagraj S, Prashanti E. School dental screening programmes for oral health.Cochrane Database Syst Rev. 2017 Dec 21;12:CD012595. doi: 10.1002/14651858.CD012595.pub2. was excluded prior to title and abstract screening, because it was an update of the following reference by the same authors in 2019: Arora A, Khattri S, Ismail NM, Kumbargere Nagraj S, Eachempati P. School dental screening programmes for oral health. Cochrane Database Syst Rev. 2019 Aug 8;8:CD012595. doi: 10.1002/14651858.CD012595.pub3.

**Rationale for exclusion of studies**

A total of 180 studies were excluded during the title and abstract screening and 45 were excluded during full text screening. The rationales for exclusion were given for each study. Only one rationale was given per study even when more than one rationales could have been applied.

Rationales for exclusion of studies:

- Assessed exclusively adverse effects
- Not an orthodontic intervention
- Not the effects of orthodontic interventions were assessed
- Empty review
- review was later updated
- Review of animal studies
- Review of laboratory studies
- Review included orthognathic surgical interventions
- Assessed exclusively patients with congenital anomalies
- Review did not assess the effect of a specific type of intervention(s), but assessed an undefined orthodontic intervention, e.g., orthodontic treatment as a whole
- Review was conducted by one operator only
- The review is about a specific outcome of an intervention, which is ambiguous and could also be an adverse effect
- A Bayesian network meta-analysis was used

**Excluded studies during the title and abstract screening (n=180) with the rationale for exclusion**

| **Journal*** | **Year** | **Reference** | **Rationale for exclusion** |
| --- | --- | --- | --- |
| 1. Cochrane library | 2019 | Mulimani P, Abas AB, Karanth L, Colombatti R, Kulkarni P. Treatment of dental and orthodontic complications in thalassaemia. Cochrane Database Syst Rev. 2019 Aug 2;8:CD012969. doi: 10.1002/14651858.CD012969.pub2. | assessed exclusively adverse effects |
| 1. Cochrane library | 2019 | Arora A, Khattri S, Ismail NM, Kumbargere Nagraj S, Eachempati P. School dental screening programmes for oral health. Cochrane Database Syst Rev. 2019 Aug 8;8:CD012595. doi: 10.1002/14651858.CD012595.pub3. | not the effects of orthodontic interventions were assessed |
| 1. Cochrane library | 2018 | Millett DT, Cunningham SJ, O'Brien KD, Benson PE, de Oliveira CM. Orthodontic treatment for deep bite and retroclined upper front teeth in children.Cochrane Database Syst Rev. 2018 Feb 1;2:CD005972. doi: 10.1002/14651858.CD005972.pub4. | empty review |
| 1. Cochrane library | 2018 | Mandall NA, Hickman J, Macfarlane TV, Mattick RC, Millett DT, Worthington HV. Adhesives for fixed orthodontic brackets.Cochrane Database Syst Rev. 2018 Apr 9;4:CD002282. doi: 10.1002/14651858.CD002282.pub2. | not the effects of orthodontic interventions were assessed |
| 1. Cochrane library | 2017 | Millett DT, Mandall NA, Mattick RC, Hickman J, Glenny AM. Adhesives for bonded molar tubes during fixed brace treatment. Cochrane Database Syst Rev. 2017 Feb 23;2:CD008236. doi: 10.1002/14651858.CD008236.pub3. | not the effects of orthodontic interventions were assessed |
| 1. Cochrane library | 2017 | Agnihotry A, Fedorowicz Z, Nasser M, Gill KS. Resorbable versus titanium plates for orthognathic surgery.Cochrane Database Syst Rev. 2017 Oct 4;10:CD006204. doi: 10.1002/14651858.CD006204.pub3. | not the effects of orthodontic interventions were assessed |
| 1. Cochrane library | 2017 | Monk AB, Harrison JE, Worthington HV, Teague A. Pharmacological interventions for pain relief during orthodontic treatment. Cochrane Database Syst Rev. 2017 Nov 28;11:CD003976. doi: 10.1002/14651858.CD003976.pub2. | not the effects of orthodontic interventions were assessed |
| 1. Cochrane library | 2016 | Ashley PF, Parekh S, Moles DR, Anand P, MacDonald LC. Preoperative analgesics for additional pain relief in children and adolescents having dental treatment. Cochrane Database Syst Rev. 2016 Aug 8;(8):CD008392. doi: 10.1002/14651858.CD008392.pub3. | not the effects of orthodontic interventions were assessed |
| 1. Cochrane library | 2016 | Ghaeminia H, Perry J, Nienhuijs ME, Toedtling V, Tummers M, Hoppenreijs TJ, Van der Sanden WJ, Mettes TG. Surgical removal versus retention for the management of asymptomatic disease-free impacted wisdom teeth. Cochrane Database Syst Rev. 2016 Aug 31;(8):CD003879. doi: 10.1002/14651858.CD003879.pub4. | not the effects of orthodontic interventions were assessed |
| 1. Cochrane library | 2016 | Carvalho FR, Lentini-Oliveira DA, Prado LB, Prado GF, Carvalho LB. Oral appliances and functional orthopaedic appliances for obstructive sleep apnoea in children. Cochrane Database Syst Rev. 2016 Oct 5;10:CD005520. | not the effects of orthodontic interventions were assessed |
| 1. Cochrane library | 2016 | Millett DT, Glenny AM, Mattick RC, Hickman J, Mandall NA. Adhesives for fixed orthodontic bands. Cochrane Database Syst Rev. 2016 Oct 25;10:CD004485. | not the effects of orthodontic interventions were assessed |
| 1. Cochrane library | 2016 | Fleming PS, Strydom H, Katsaros C, MacDonald L, Curatolo M, Fudalej P, Pandis N. Non-pharmacological interventions for alleviating pain during orthodontic treatment. Cochrane Database Syst Rev. 2016 Dec 23;12:CD010263. doi: 10.1002/14651858.CD010263.pub2. | not the effects of orthodontic interventions were assessed |
| 1. Cochrane library | 2015 | Ahangari Z, Nasser M, Mahdian M, Fedorowicz Z, Marchesan MA. Interventions for the management of external root resorption. Cochrane Database Syst Rev. 2015 Nov 24;(11):CD008003. doi: 10.1002/14651858.CD008003.pub3.(discuss met Reint: moeten we deze wel includeren) | assessed exclusively adverse effects |
| 1. Cochrane library | 2013 | Belmonte FM, Macedo CR, Day PF, Saconato H, Fernandes Moça Trevisani V. Interventions for treating traumatised permanent front teeth: luxated (dislodged) teeth.Cochrane Database Syst Rev. 2013 Apr 30;(4):CD006203. doi: 10.1002/14651858.CD006203.pub2. | empty review |
| 1. Cochrane library | 2013 | Yu Y, Sun J, Lai W, Wu T, Koshy S, Shi Z. Interventions for managing relapse of the lower front teeth after orthodontic treatment. Cochrane Database Syst Rev. 2013 Sep 6;(9):CD008734. doi: 10.1002/14651858.CD008734.pub2. | empty review |
| 1. Cochrane library | 2013 | Hu H, Li C, Li F, Chen J, Sun J, Zou S, Sandham A, Xu Q, Riley P, Ye Q. Enamel etching for bonding fixed orthodontic braces. Cochrane Database Syst Rev. 2013 Nov 25;(11):CD005516. doi: 10.1002/14651858.CD005516.pub2. | not the effects of orthodontic interventions were assessed |
| 1. Cochrane library | 2013 | Benson PE, Parkin N, Dyer F, Millett DT, Furness S, Germain P. Fluorides for the prevention of early tooth decay (demineralised white lesions) during fixed brace treatment. Cochrane Database Syst Rev. 2013 Dec 12;(12):CD003809. doi: 10.1002/14651858.CD003809.pub3. | not the effects of orthodontic interventions were assessed |
| 1. EJO | 2021 | Alrashed M, Alqerban A. The relationship between malocclusion and oral health-related quality of life among adolescents: a systematic literature review and meta-analysis. Eur J Orthod. 2021 Apr 3;43(2):173-183. doi: 10.1093/ejo/cjaa051. PMID: 33009547. | not the effects of orthodontic interventions were assessed |
| 1. EJO | 2021 | Mulier D, Gaitán Romero L, Führer A, Martin C, Shujaat S, Shaheen E, Politis C, Jacobs R. Long-term dental stability after orthognathic surgery: a systematic review. Eur J Orthod. 2021 Jan 29;43(1):104-112. doi: 10.1093/ejo/cjaa022. PMID: 32901268. | review included orthognathic surgical interventions |
| 1. EJO | 2021 | Palikaraki G, Makrygiannakis MA, Zafeiriadis AA, Benetou V, Sanoudos M, Bitsanis I, Tsolakis AI. The effect of facemask in patients with unilateral cleft lip and palate: a systematic review and meta-analysis. Eur J Orthod. 2021 Jan 29;43(1):69-79. doi: 10.1093/ejo/cjaa027. PMID: 32274494. | assessed exclusively patients with congenital anomalies |
| 1. EJO | 2021 | Gandhi V, Mehta S, Gauthier M, Mu J, Kuo CL, Nanda R, Yadav S. Comparison of external apical root resorption with clear aligners and pre-adjusted edgewise appliances in non-extraction cases: a systematic review and meta-analysis. Eur J Orthod. 2021 Jan 29;43(1):15-24. doi: 10.1093/ejo/cjaa013. PMID: 32077935; PMCID: PMC7846172. | assessed exclusively adverse effects |
| 1. EJO | 2020 | Stucki S, Gkantidis N. Assessment of techniques used for superimposition of maxillary and mandibular 3D surface models to evaluate tooth movement: a systematic review. Eur J Orthod. 2020 Nov 3;42(5):559-570. doi: 10.1093/ejo/cjz075. PMID: 31742598. | not an orthodontic intervention |
| 1. EJO | 2020 | Rodrigues AS, Antunes LS, Pinheiro LHM, Guimarães LS, Calansans-Maia JA, Küchler EC, Antunes LAA. Is dental agenesis associated with craniofacial morphology pattern? A systematic review and meta-analysis. Eur J Orthod. 2020 Nov 3;42(5):534-543. doi: 10.1093/ejo/cjz087. PMID: 31783403. | not an orthodontic intervention |
| 1. EJO | 2020 | Kaklamanos EG, Makrygiannakis MA, Athanasiou AE. Does medication administration affect the rate of orthodontic tooth movement and root resorption development in humans? A systematic review. Eur J Orthod. 2020 Sep 11;42(4):407-414. doi: 10.1093/ejo/cjz063. PMID: 31421637. | not the effects of orthodontic interventions were assessed |
| 1. EJO | 2020 | de Araujo CM, Schroder AGD, de Araujo BMM, Cavalcante-Leão BL, Stechman-Neto J, Zeigelboim BS, Santos RS, Guariza-Filho O. Impact of orthodontic-surgical treatment on quality of life: a meta-analysis. Eur J Orthod. 2020 Jun 23;42(3):281-289. doi: 10.1093/ejo/cjz093. PMID: 31784741. | review included orthognathic surgical interventions |
| 1. EJO | 2020 | Papadimitriou A, Kakali L, Pazera P, Doulis I, Kloukos D. Social media and orthodontic treatment from the patient's perspective: a systematic review. Eur J Orthod. 2020 Jun 23;42(3):231-241. doi: 10.1093/ejo/cjz029. PMID: 31107943. | not the effects of orthodontic interventions were assessed |
| 1. EJO | 2020 | Zymperdikas VF, Yavropoulou MP, Kaklamanos EG, Papadopoulos MA. Effects of systematic bisphosphonate use in patients under orthodontic treatment: a systematic review. Eur J Orthod. 2020 Jan 27;42(1):60-71. doi: 10.1093/ejo/cjz021. PMID: 31009953. | not an orthodontic intervention |
| 1. EJO | 2019 | Sardana D, Manchanda S, Ekambaram M, Yang Y, McGrath CP, Yiu CKY. Effectiveness of self-applied topical fluorides against enamel white spot lesions from multi-bracketed fixed orthodontic treatment: a systematic review. Eur J Orthod. 2019 Nov 15;41(6):661-668. doi: 10.1093/ejo/cjz015. Erratum in: Eur J Orthod. 2019 Nov 15;41(6):669. PMID: 31112229. | not an orthodontic intervention |
| 1. EJO | 2019 | Makrygiannakis MA, Kaklamanos EG, Athanasiou AE. Does long-term use of pain relievers have an impact on the rate of orthodontic tooth movement? A systematic review of animal studies. Eur J Orthod. 2019 Sep 21;41(5):468-477. doi: 10.1093/ejo/cjy079. PMID: 30590549. | review of animal studies |
| 1. EJO | 2019 | Kakali L, Alharbi M, Pandis N, Gkantidis N, Kloukos D. Success of palatal implants or mini-screws placed median or paramedian for the reinforcement of anchorage during orthodontic treatment: a systematic review. Eur J Orthod. 2019 Jan 23;41(1):9-20. doi: 10.1093/ejo/cjy015. | not the effects of orthodontic interventions were assessed |
| 1. EJO | 2019 | Samandara A, Papageorgiou SN, Ioannidou-Marathiotou I, Kavvadia-Tsatala S, Papadopoulos MA. Evaluation of orthodontically induced external root resorption following orthodontic treatment using cone beam computed tomography (CBCT): a systematic review and meta-analysis. Eur J Orthod. 2019 Jan 23;41(1):67-79. doi: 10.1093/ejo/cjy027. | assessed exclusively adverse effects |
| 1. EJO | 2019 | Bartolucci ML, Bortolotti F, Martina S2, Corazza G, Michelotti A, Alessandri-Bonetti G. Dental and skeletal long-term side effects of mandibular advancement devices in obstructive sleep apnea patients: a systematic review with meta-regression analysis. Eur J Orthod. 2019 Jan 23;41(1):89-100. doi: 10.1093/ejo/cjy036. | not the effects of orthodontic interventions were assessed |
| 1. EJO | 2019 | Mohammed H, Rizk MZ, Wafaie K, Ulhaq A, Almuzian M. Reminders improve oral hygiene and adherence to appointments in orthodontic patients: a systematic review and meta-analysis. Eur J Orthod. 2019 Mar 29;41(2):204-213. doi: 10.1093/ejo/cjy045. | not the effects of orthodontic interventions were assessed |
| 1. EJO | 2019 | Makrygiannakis MA, Kaklamanos EG, Athanasiou AE. Effects of systemic medication on root resorption associated with orthodontic tooth movement: a systematic review of animal studies. Eur J Orthod. 2019 Aug 8;41(4):346-359. doi: 10.1093/ejo/cjy048. | review of animal studies |
| 1. EJO | 2019 | De Grauwe A, Ayaz I, Shujaat S, Dimitrov S, Gbadegbegnon L, Vande Vannet B, Jacobs R. CBCT in orthodontics: a systematic review on justification of CBCT in a paediatric population prior to orthodontic treatment. Eur J Orthod. 2019 Aug 8;41(4):381-389. doi: 10.1093/ejo/cjy066. | not the effects of orthodontic interventions were assessed |
| 1. EJO | 2018 | Chen J, Wan J, You L. Speech and orthodontic appliances: a systematic literature review.Eur J Orthod. 2018 Jan 23;40(1):29-36. doi: 10.1093/ejo/cjx023. | assessed exclusively adverse effects |
| 1. EJO | 2018 | Alyammahi AS, Kaklamanos EG, Athanasiou AE. Effectiveness of extraction of primary canines for interceptive management of palatally displaced permanent canines: a systematic review and meta-analysis.Eur J Orthod. 2018 Apr 6;40(2):149-156. doi: 10.1093/ejo/cjx042. | not the effects of orthodontic interventions were assessed |
| 1. EJO | 2018 | Lo Giudice A, Barbato E, Cosentino L, Ferraro CM, Leonardi R Alveolar bone changes after rapid maxillary expansion with tooth-born appliances: a systematic review.Eur J Orthod. 2018 May 25;40(3):296-303. doi: 10.1093/ejo/cjx057. | assessed exclusively adverse effects |
| 1. EJO | 2018 | Haugland L, Kristensen KD, Lie SA, Vandevska-Radunovic V. The effect of biologic factors and adjunctive therapies on orthodontically induced inflammatory root resorption: a systematic review and meta-analysis.Eur J Orthod. 2018 May 25;40(3):326-336. doi: 10.1093/ejo/cjy003. | assessed exclusively adverse effects |
| 1. EJO | 2018 | Alharbi F, Almuzian M, Bearn D. Miniscrews failure rate in orthodontics: systematic review and meta-analysis.Eur J Orthod. 2018 Sep 28;40(5):519-530. doi: 10.1093/ejo/cjx093. | assessed exclusively adverse effects |
| 1. EJO | 2018 | Grisar K, Chaabouni D, Romero LPG, Vandendriessche T, Politis C, Jacobs R. Autogenous transalveolar transplantation of maxillary canines: a systematic review and meta-analysis.Eur J Orthod. 2018 Nov 30;40(6):608-616. doi: 10.1093/ejo/cjy026. | not an orthodontic intervention |
| 1. EJO | 2018 | Dumbryte I, Vebriene J, Linkeviciene L, Malinauskas M. Enamel microcracks in the form of tooth damage during orthodontic debonding: a systematic review and meta-analysis of in vitro studies.Eur J Orthod. 2018 Nov 30;40(6):636-648. doi: 10.1093/ejo/cjx102. | review of laboratory studies |
| 1. EJO | 2018 | Makrygiannakis MA, Kaklamanos EG, Athanasiou AE. Does common prescription medication affect the rate of orthodontic tooth movement? A systematic review.Eur J Orthod. 2018 Nov 30;40(6):649-659. doi: 10.1093/ejo/cjy001. | review of animal studies |
| 1. EJO | 2017 | Castroflorio T, Bargellini A, Rossini G, Cugliari G, Deregibus A. Sleep bruxism in adolescents: a systematic literature review of related risk factors. Eur J Orthod. 2017 Feb;39(1):61-68. doi: 10.1093/ejo/cjw012. | not an orthodontic intervention |
| 1. EJO | 2017 | Höchli D, Hersberger-Zurfluh M, Papageorgiou SN, Eliades T. Interventions for orthodontically induced white spot lesions: a systematic review and meta-analysis. Eur J Orthod. 2017 Apr 1;39(2):122-133. doi: 10.1093/ejo/cjw065. | not an orthodontic intervention |
| 1. EJO | 2017 | Sonesson M, Bergstrand F, Gizani S, Twetman S. Management of post-orthodontic white spot lesions: an updated systematic review. Eur J Orthod. 2017 Apr 1;39(2):116-121. doi: 10.1093/ejo/cjw023. | not an orthodontic intervention |
| 1. EJO | 2017 | Dalessandri D, Parrini S, Rubiano R, Gallone D, Migliorati M. Impacted and transmigrant mandibular canines incidence, aetiology, and treatment: a systematic review. Eur J Orthod. 2017 Apr 1;39(2):161-169. doi: 10.1093/ejo/cjw027. | not an orthodontic intervention |
| 1. EJO | 2017 | Yi J, Ge M, Li M, Li C, Li Y, Li X, Zhao Z. Comparison of the success rate between self-drilling and self-tapping miniscrews: a systematic review and meta-analysis. Eur J Orthod. 2017 Jun 1;39(3):287-293. doi: 10.1093/ejo/cjw036. | not the effects of orthodontic interventions were assessed |
| 1. EJO | 2017 | Mousoulea S, Kloukos D, Sampaziotis D, Vogiatzi T, Eliades T. Condylar resorption in orthognathic patients after mandibular bilateral sagittal split osteotomy: a systematic review. Eur J Orthod. 2017 Jun 1;39(3):294-309. doi: 10.1093/ejo/cjw045. | assessed exclusively adverse effects |
| 1. EJO | 2017 | Zimmerman JN, Lee J, Pliska BT. Reliability of upper pharyngeal airway assessment using dental CBCT: a systematic review. Eur J Orthod. 2017 Oct 1;39(5):489-496. doi: 10.1093/ejo/cjw079. | not an orthodontic intervention |
| 1. EJO | 2017 | Becking BE, Verweij JP, Kalf-Scholte SM, Valkenburg C, Bakker EWP, van Merkesteyn JPR. Impact of adenotonsillectomy on the dentofacial development of obstructed children: a systematic review and meta-analysis. Eur J Orthod. 2017 Oct 1;39(5):509-518. doi: 10.1093/ejo/cjx005. | not an orthodontic intervention |
| 1. EJO | 2017 | Sandhu SS, Piepho HP, Khehra HS. Comparing the effectiveness profile of pharmacological interventions used for orthodontic pain relief: an arm-based multilevel network meta-analysis of longitudinal data. Eur J Orthod. 2017 Nov 30;39(6):601-614. doi: 10.1093/ejo/cjw088. | not an orthodontic intervention |
| 1. EJO | 2016 | Rakhshan V, Rakhshan H. Meta-analysis and systematic review of the number of non-syndromic congenitally missing permanent teeth per affected individual and its influencing factors. Eur J Orthod. 2016 Apr;38(2):170-7. doi: 10.1093/ejo/cjv008. | not an orthodontic intervention |
| 1. EJO | 2016 | Goracci C, Franchi L, Vichi A, Ferrari M3. Accuracy, reliability, and efficiency of intraoral scanners for full-arch impressions: a systematic review of the clinical evidence. Eur J Orthod. 2016 Aug;38(4):422-8. doi: 10.1093/ejo/cjv077. | not an orthodontic intervention |
| 1. EJO | 2016 | Aragón ML, Pontes LF, Bichara LM, Flores-Mir C, Normando D. Validity and reliability of intraoral scanners compared to conventional gypsum models measurements: a systematic review. Eur J Orthod. 2016 Aug;38(4):429-34. doi: 10.1093/ejo/cjw033. (geen PDF) | not an orthodontic intervention |
| 1. EJO | 2016 | Iodice G, Danzi G, Cimino R, Paduano S, Michelotti A. Association between posterior crossbite, skeletal, and muscle asymmetry: a systematic review. Eur J Orthod. 2016 Dec;38(6):638-651. | not an orthodontic intervention |
| 1. EJO | 2015 | Dimberg L, Arnrup K, Bondemark L. The impact of malocclusion on the quality of life among children and adolescents: a systematic review of quantitative studies. Eur J Orthod. 2015 Jun;37(3):238-47. doi: 10.1093/ejo/cju046. | not an orthodontic intervention |
| 1. EJO | 2015 | Migliorati M, Isaia L, Cassaro A, Rivetti A, Silvestrini-Biavati F, Gastaldo L, Piccardo I, Dalessandri D, Silvestrini-Biavati A. Efficacy of professional hygiene and prophylaxis on preventing plaque increase in orthodontic patients with multibracket appliances: a systematic review. Eur J Orthod. 2015 Jun;37(3):297-307. doi: 10.1093/ejo/cju044. | not an orthodontic intervention |
| 1. EJO | 2015 | Nazarali N, Altalibi M, Nazarali S, Major MP, Flores-Mir C, Major PW. Mandibular advancement appliances for the treatment of paediatric obstructive sleep apnea: a systematic review. Eur J Orthod. 2015 Dec;37(6):618-26. doi: 10.1093/ejo/cju101. | not an orthodontic intervention |
| 1. EJO | 2014 | Gomes Lde C, Horta KO, Gonçalves JR, Santos-Pinto AD. Systematic review: craniocervical posture and craniofacial morphology. Eur J Orthod. 2014 Feb;36(1):55-66. doi: 10.1093/ejo/cjt004. | not an orthodontic intervention |
| 1. EJO | 2014 | Dalessandri D, Salgarello S, Dalessandri M, Lazzaroni E, Piancino M, Paganelli C, Maiorana C, Santoro F. Determinants for success rates of temporary anchorage devices in orthodontics: a meta-analysis (n > 50). Eur J Orthod. 2014 Jun;36(3):303-13. doi: 10.1093/ejo/cjt049. | not the effects of orthodontic interventions were assessed |
| 1. EJO | 2014 | Kloukos D, Eliades T, Sculean A, Katsaros C. Indication and timing of soft tissue augmentation at maxillary and mandibular incisors in orthodontic patients. A systematic review.Eur J Orthod. 2014 Aug;36(4):442-9. doi: 10.1093/ejo/cjt073. | not an orthodontic intervention |
| 1. EJO | 2014 | Winsauer H, Vlachojannis C, Bumann A, Vlachojannis J, Chrubasik S. Paramedian vertical palatal bone height for mini-implant insertion: a systematic review. Eur J Orthod. 2014 Oct;36(5):541-9. doi: 10.1093/ejo/cjs068. | not an orthodontic intervention |
| 1. EJO | 2014 | Forst D, Nijjar S, Khaled Y, Lagravere M, Flores-Mir C. Radiographic assessment of external root resorption associated with jackscrew-based maxillary expansion therapies: a systematic review. Eur J Orthod. 2014 Oct;36(5):576-85. doi: 10.1093/ejo/cjt090. | assessed exclusively adverse effects |
| 1. EJO | 2013 | Livas C, Delli K. Subjective and objective perception of orthodontic treatment need: a systematic review.Eur J Orthod. 2013 Jun;35(3):347-53. doi: 10.1093/ejo/cjr142. | not an orthodontic intervention |
| 1. EJO | 2013 | Alves M Jr, Baratieri C, Mattos CT, Araújo MT, Maia LC. Root repair after contact with mini-implants: systematic review of the literature.Eur J Orthod. 2013 Aug;35(4):491-9. doi: 10.1093/ejo/cjs025. Epub 2012 Apr 26. | not an orthodontic intervention |
| 1. EJO | 2013 | Perinetti G, Primozic J, Manfredini D, Di Lenarda R, Contardo L. The diagnostic potential of static body-sway recording in orthodontics: a systematic review. Eur J Orthod. 2013 Oct;35(5):696-705. doi: 10.1093/ejo/cjs085. | not an orthodontic intervention |
| 1. EJO | 2013 | Iodice G, Danzi G, Cimino R, Paduano S, Michelotti A. Association between posterior crossbite, masticatory muscle pain, and disc displacement: a systematic review. Eur J Orthod. 2013 Dec;35(6):737-44. doi: 10.1093/ejo/cjt024. | not an orthodontic intervention |
| 1. EJO | 2013 | Altalibi M, Saltaji H, Edwards R, Major PW, Flores-Mir C. Indices to assess malocclusions in patients with cleft lip and palate. Eur J Orthod. 2013 Dec;35(6):772-82. doi: 10.1093/ejo/cjt009. | not an orthodontic intervention |
| 1. EJO | 2013 | Walker SL, Tieu LD, Flores-Mir C. Radiographic comparison of the extent of orthodontically induced external apical root resorption in vital and root-filled teeth: a systematic review. Eur J Orthod. 2013 Dec;35(6):796-802. doi: 10.1093/ejo/cjs101. | assessed exclusively adverse effects |
| 1. EJO | 2011 | Fudalej P, Dragan M, Wedrychowska-Szulc B. Prediction of the outcome of orthodontic treatment of Class III malocclusions--a systematic review. Eur J Orthod. 2011 Apr;33(2):190-7. doi: 10.1093/ejo/cjq052. | not an orthodontic intervention |
| 1. EJO | 2011 | Ahrens A, McGrath C, Hägg U. A systematic review of the efficacy of oral appliance design in the management of obstructive sleep apnoea. Eur J Orthod. 2011 Jun;33(3):318-24. doi: 10.1093/ejo/cjq079. | not an orthodontic intervention |
| 1. AJODO | 2021 | Malekshoar M, Malekshoar M, Javanshir B. Challenges, limitations, and solutions for orthodontists during the coronavirus pandemic: A review. Am J Orthod Dentofacial Orthop. 2021 Jan;159(1):e59-e71. doi: 10.1016/j.ajodo.2020.09.009. Epub 2020 Oct 19. PMID: 33223376; PMCID: PMC7571895. | not the effects of orthodontic interventions were assessed |
| 1. AJODO | 2020 | Tepedino M, Cattaneo PM, Niu X, Cornelis MA. Interradicular sites and cortical bone thickness for miniscrew insertion: A systematic review with meta-analysis. Am J Orthod Dentofacial Orthop. 2020 Dec;158(6):783-798.e20. doi: 10.1016/j.ajodo.2020.05.011. Epub 2020 Oct 16. Erratum in: Am J Orthod Dentofacial Orthop. 2021 Jun;159(6):711. PMID: 33077369. | not the effects of orthodontic interventions were assessed |
| 1. AJODO | 2020 | ElShehaby M, Mofti B, Montasser MA, Bearn D. Powered vs manual tooth brushing in patients with fixed orthodontic appliances: A systematic review and meta-analysis. Am J Orthod Dentofacial Orthop. 2020 Nov;158(5):639-649. doi: 10.1016/j.ajodo.2020.04.018. Epub 2020 Sep 17. PMID: 32951930. | not an orthodontic intervention |
| 1. AJODO | 2020 | Macey R, Thiruvenkatachari B, O'Brien K, Batista KBSL. Do malocclusion and orthodontic treatment impact oral health? A systematic review and meta-analysis. Am J Orthod Dentofacial Orthop. 2020 Jun;157(6):738-744.e10. doi: 10.1016/j.ajodo.2020.01.015. PMID: 32487303. | review did not assess the effect of a specific type of intervention(s), but assessed an undefined orthodontic intervention, e.g., orthodontic treatment as a whole |
| 1. AJODO | 2020 | Arn ML, Dritsas K, Pandis N, Kloukos D. The effects of fixed orthodontic retainers on periodontal health: A systematic review. Am J Orthod Dentofacial Orthop. 2020 Feb;157(2):156-164.e17. doi: 10.1016/j.ajodo.2019.10.010. PMID: 32005466. | assessed exclusively adverse effects |
| 1. AJODO | 2019 | Vásquez-Cárdenas J, Zapata-Noreña Ó, Carvajal-Flórez Á, Barbosa-Liz DM, Giannakopoulos NN, Faggion CM Jr. Systematic reviews in orthodontics: Impact of the PRISMA for Abstracts checklist on completeness of reporting. Am J Orthod Dentofacial Orthop. 2019 Oct;156(4):442-452.e12. doi: 10.1016/j.ajodo.2019.05.009. PMID: 31582116. | not an orthodontic intervention |
| 1. AJODO | 2019 | Barretto Dos Santos Lopes Batista K, Thiruvenkatachari B, O'Brien K. Intention-to-treat analysis: Are we managing dropouts and missing data properly in research on orthodontic treatment? A systematic review. Am J Orthod Dentofacial Orthop. 2019 Jan;155(1):19-27.e3. doi: 10.1016/j.ajodo.2018.08.013. | not an orthodontic intervention |
| 1. AJODO | 2019 | Currell SD, Liaw A, Blackmore Grant PD, Esterman A4, Nimmo A. Orthodontic mechanotherapies and their influence on external root resorption: A systematic review. Am J Orthod Dentofacial Orthop. 2019 Mar;155(3):313-329. doi: 10.1016/j.ajodo.2018.10.015. | assessed exclusively adverse effects |
| 1. AJODO | 2019 | Yi J, Lu W, Xiao J, Li X, Li Y, Zhao Z. Effect of conventional combined orthodontic-surgical treatment on oral health-related quality of life: A systematic review and meta-analysis. Am J Orthod Dentofacial Orthop. 2019 Jul;156(1):29-43.e5. doi: 10.1016/j.ajodo.2019.03.008. | review included orthognathic surgical interventions |
| 1. AJODO | 2018 | Al-Jewair T, Stellrecht E, Lewandowski L, Chakaki R. American Association of Orthodontists Foundation Craniofacial Growth Legacy Collection in the orthodontic literature-use and trends: A systematic review.Am J Orthod Dentofacial Orthop. 2018 Jan;153(1):15-25.e10. doi: 10.1016/j.ajodo.2017.07.015. | not an orthodontic intervention |
| 1. AJODO | 2018 | Barber S, Bekker HL, Meads D, Pavitt S, Khambay B. Identification and appraisal of outcome measures used to evaluate hypodontia care: A systematic review.Am J Orthod Dentofacial Orthop. 2018 Feb;153(2):184-194.e18. doi: 10.1016/j.ajodo.2017.10.010. | not an orthodontic intervention |
| 1. AJODO | 2017 | Eslami E, Barkhordar H, Abramovitch K, Kim J, Masoud MI. Cone-beam computed tomography vs conventional radiography in visualization of maxillary impacted-canine localization: A systematic review of comparative studies. Am J Orthod Dentofacial Orthop. 2017 Feb;151(2):248-258. doi: 10.1016/j.ajodo.2016.07.018. | not an orthodontic intervention |
| 1. AJODO | 2017 | Al-Moghrabi D, Salazar FC, Pandis N, Fleming PS. Compliance with removable orthodontic appliances and adjuncts: A systematic review and meta-analysis. Am J Orthod Dentofacial Orthop. 2017 Jul;152(1):17-32. doi: 10.1016/j.ajodo.2017.03.019. | not the effects of orthodontic interventions were assessed |
| 1. AJODO | 2017 | Al Makhmari SA, Kaklamanos EG, Athanasiou AE. Short-term and long-term effectiveness of powered toothbrushes in promoting periodontal health during orthodontic treatment: A systematic review and meta-analysis. Am J Orthod Dentofacial Orthop. 2017 Dec;152(6):753-766.e7. doi: 10.1016/j.ajodo.2017.09.003. | not an orthodontic intervention |
| 1. AJODO | 2016 | Rossini G, Parrini S, Castroflorio T, Deregibus A, Debernardi CL. Diagnostic accuracy and measurement sensitivity of digital models for orthodontic purposes: A systematic review. Am J Orthod Dentofacial Orthop. 2016 Feb;149(2):161-70. doi: 10.1016/j.ajodo.2015.06.029. | not an orthodontic intervention |
| 1. AJODO | 2016 | Peiró-Guijarro MA, Guijarro-Martínez R, Hernández-Alfaro F. Surgery first in orthognathic surgery: A systematic review of the literature. Am J Orthod Dentofacial Orthop. 2016 Apr;149(4):448-62. doi: 10.1016/j.ajodo.2015.09.022. | not an orthodontic intervention |
| 1. AJODO | 2016 | Incerti-Parenti S, Checchi V, Ippolito DR, Gracco A, Alessandri-Bonetti G. Periodontal status after surgical-orthodontic treatment of labially impacted canines with different surgical techniques: A systematic review. Am J Orthod Dentofacial Orthop. 2016 Apr;149(4):463-72. doi: 10.1016/j.ajodo.2015.10.019. | not an orthodontic intervention |
| 1. AJODO | 2016 | Ata-Ali F, Ata-Ali J, Ferrer-Molina M, Cobo T, De Carlos F, Cobo J. Adverse effects of lingual and buccal orthodontic techniques: A systematic review and meta-analysis. Am J Orthod Dentofacial Orthop. 2016 Jun;149(6):820-9. doi: 10.1016/j.ajodo.2015.11.031. | assessed exclusively adverse effects |
| 1. AJODO | 2016 | Sandhu SS, Cheema MS, Khehra HS. Comparative effectiveness of pharmacologic and nonpharmacologic interventions for orthodontic pain relief at peak pain intensity: A Bayesian network meta-analysis. Am J Orthod Dentofacial Orthop. 2016 Jul;150(1):13-32. doi: 10.1016/j.ajodo.2015.12.025. | not an orthodontic intervention |
| 1. AJODO | 2016 | Cadenas-Perula M, Yañez-Vico RM, Solano-Reina E, Iglesias-Linares A. Effectiveness of biologic methods of inhibiting orthodontic tooth movement in animal studies. Am J Orthod Dentofacial Orthop. 2016 Jul;150(1):33-48. doi: 10.1016/j.ajodo.2016.01.015. | review of animal studies |
| 1. AJODO | 2016 | Parrini S, Rossini G, Castroflorio T, Fortini A, Deregibus A, Debernardi C. Laypeople's perceptions of frontal smile esthetics: A systematic review. Am J Orthod Dentofacial Orthop. 2016 Nov;150(5):740-750. doi: 10.1016/j.ajodo.2016.06.022. | not the effects of orthodontic interventions were assessed |
| 1. AJODO | 2015 | Roscoe MG, Meira JB, Cattaneo PM. Association of orthodontic force system and root resorption: A systematic review. Am J Orthod Dentofacial Orthop. 2015 May;147(5):610-26. doi: 10.1016/j.ajodo.2014.12.026. | assessed exclusively adverse effects |
| 1. AJODO | 2014 | Afrand M, Ling CP, Khosrotehrani S, Flores-Mir C, Lagravère-Vich MO. Anterior cranial-base time-related changes: A systematic review. Am J Orthod Dentofacial Orthop. 2014 Jul;146(1):21-32.e6. doi: 10.1016/j.ajodo.2014.03.019. | not an orthodontic intervention |
| 1. AJODO | 2014 | Tsichlaki A, O'Brien K. Do orthodontic research outcomes reflect patient values? A systematic review of randomized controlled trials involving children. Am J Orthod Dentofacial Orthop. 2014 Sep;146(3):279-85. doi: 10.1016/j.ajodo.2014.05.022. | not the effects of orthodontic interventions were assessed |
| 1. AJODO | 2013 | Katyal V, Pamula Y, Martin AJ, Daynes CN, Kennedy JD, Sampson WJ. Craniofacial and upper airway morphology in pediatric sleep-disordered breathing: Systematic review and meta-analysis.Am J Orthod Dentofacial Orthop. 2013 Jan;143(1):20-30.e3. doi: 10.1016/j.ajodo.2012.08.021. | not an orthodontic intervention |
| 1. AJODO | 2013 | Chen H, Liu X, Dai J, Jiang Z, Guo T, Ding Y. Effect of remineralizing agents on white spot lesions after orthodontic treatment: a systematic review. Am J Orthod Dentofacial Orthop. 2013 Mar;143(3):376-382.e3. doi: 10.1016/j.ajodo.2012.10.013. | not an orthodontic intervention |
| 1. AJODO | 2013 | Fleming PS, Eliades T, Katsaros C, Pandis N. Curing lights for orthodontic bonding: a systematic review and meta-analysis. Am J Orthod Dentofacial Orthop. 2013 Apr;143(4 Suppl):S92-103. doi: 10.1016/j.ajodo.2012.07.018. | not an orthodontic intervention |
| 1. AJODO | 2013 | Kloukos D, Pandis N, Eliades T. Bisphenol-A and residual monomer leaching from orthodontic adhesive resins and polycarbonate brackets: a systematic review. Am J Orthod Dentofacial Orthop. 2013 Apr;143(4 Suppl):S104-12.e1-2. doi: 10.1016/j.ajodo.2012.11.015. | not an orthodontic intervention |
| 1. AJODO | 2013 | Hua F, He H, Ngan P, Bouzid W. Prevalence of peg-shaped maxillary permanent lateral incisors: A meta-analysis. Am J Orthod Dentofacial Orthop. 2013 Jul;144(1):97-109. doi: 10.1016/j.ajodo.2013.02.025. | not an orthodontic intervention |
| 1. AJODO | 2012 | Fleming PS, Johal A, Pandis N. Self-etch primers and conventional acid-etch technique for orthodontic bonding: a systematic review and meta-analysis. Am J Orthod Dentofacial Orthop. 2012 Jul;142(1):83-94. doi: 10.1016/j.ajodo.2012.02.023. | not an orthodontic intervention |
| 1. AJODO | 2012 | Hafez HS, Shaarawy SM, Al-Sakiti AA, Mostafa YA. Dental crowding as a caries risk factor: a systematic review. Am J Orthod Dentofacial Orthop. 2012 Oct;142(4):443-50. doi: 10.1016/j.ajodo.2012.04.018. | not an orthodontic intervention |
| 1. AJODO | 2012 | Meursinge Reynders RA, Ronchi L, Ladu L, van Etten-Jamaludin F, Bipat S. Insertion torque and success of orthodontic mini-implants: a systematic review. Am J Orthod Dentofacial Orthop. 2012 Nov;142(5):596-614.e5. doi: 10.1016/j.ajodo.2012.06.013. | not the effects of orthodontic interventions were assessed |
| 1. AJODO | 2012 | Papageorgiou SN, Zogakis IP, Papadopoulos MA. Failure rates and associated risk factors of orthodontic miniscrew implants: a meta-analysis. Am J Orthod Dentofacial Orthop. 2012 Nov;142(5):577-595.e7. doi: 10.1016/j.ajodo.2012.05.016. | not the effects of orthodontic interventions were assessed |
| 1. AJODO | 2010 | Crismani AG, Bertl MH, Celar AG, Bantleon HP, Burstone CJ. Miniscrews in orthodontic treatment: review and analysis of published clinical trials. Am J Orthod Dentofacial Orthop. 2010 Jan;137(1):108-13. doi: 10.1016/j.ajodo.2008.01.027. | not the effects of orthodontic interventions were assessed |
| 1. AJODO | 2010 | Weltman B, Vig KW, Fields HW, Shanker S, Kaizar EE. Root resorption associated with orthodontic tooth movement: a systematic review. Am J Orthod Dentofacial Orthop. 2010 Apr;137(4):462-76; discussion 12A. doi: 10.1016/j.ajodo.2009.06.021. | assessed exclusively adverse effects |
| 1. AJODO | 2010 | Finnema KJ, Ozcan M, Post WJ, Ren Y, Dijkstra PU. In-vitro orthodontic bond strength testing: a systematic review and meta-analysis. Am J Orthod Dentofacial Orthop. 2010 May;137(5):615-622.e3. doi: 10.1016/j.ajodo.2009.12.021. | review of laboratory studies |
| 1. AJODO | 2010 | Rogers S, Chadwick B, Treasure E. Fluoride-containing orthodontic adhesives and decalcification in patients with fixed appliances: a systematic review. Am J Orthod Dentofacial Orthop. 2010 Oct;138(4):390.e1-8; discussion 390-1. doi: 10.1016/j.ajodo.2010.02.025. | not an orthodontic intervention |
| 1. AJODO | 2010 | Ahrens A, McGrath C, Hägg U. Subjective efficacy of oral appliance design features in the management of obstructive sleep apnea: a systematic review. Am J Orthod Dentofacial Orthop. 2010 Nov;138(5):559-76. doi: 10.1016/j.ajodo.2010.01.030. | not an orthodontic intervention |
| 1. AJODO | 2009 | Al-Riyami S, Moles DR, Cunningham SJ. Orthognathic treatment and temporomandibular disorders: a systematic review. Part 1. A new quality-assessment technique and analysis of study characteristics and classifications. Am J Orthod Dentofacial Orthop. 2009 Nov;136(5):624.e1-15; discussion 624-5. doi: 10.1016/j.ajodo.2009.02.021. | not the effects of orthodontic interventions were assessed |
| 1. AJODO | 2009 | Al-Riyami S, Cunningham SJ, Moles DR. Orthognathic treatment and temporomandibular disorders: a systematic review. Part 2. Signs and symptoms and meta-analyses. Am J Orthod Dentofacial Orthop. 2009 Nov;136(5):626.e1-16, discussion 626-7. doi: 10.1016/j.ajodo.2009.02.022. | not the effects of orthodontic interventions were assessed |
| 1. AO | 2021 | Santana LG, Avelar K, Marques LS. Association between arch perimeter management and the occurrence of mandibular second molar eruption disturbances. Angle Orthod. 2021 Jul 1;91(4):544-554. doi: 10.2319/091720-799.1. PMID: 33587114; PMCID: PMC8259765. | not the effects of orthodontic interventions were assessed |
| 1. AO | 2021 | Li Z, Zhou J, Chen S. The effectiveness of locally injected platelet-rich plasma on orthodontic tooth movement acceleration. Angle Orthod. 2021 May 1;91(3):391-398. doi: 10.2319/061320-544.1. PMID: 33566068; PMCID: PMC8084470. | review of animal studies |
| 1. AO | 2021 | Veginadu P, Tavva SR, Muddada V, Gorantla S. Effect of pharmacological agents on relapse following orthodontic tooth movement. Angle Orthod. 2020 Jul 1;90(4):598-606. doi: 10.2319/092619-613.1. PMID: 33378496; PMCID: PMC8028453. | review of animal studies |
| 1. AO | 2020 | Yao K, Zhu G, Chen M, Zhang B, Wu Y, Li P. Effect of surgery-first orthognathic approach on oral health-related quality of life. Angle Orthod. 2020 Sep 1;90(5):723-733. doi: 10.2319/112619-749.1. Erratum in: Angle Orthod. 2021 Mar 1;91(2):279-281. PMID: 33378482; PMCID: PMC8032263. | review included orthognathic surgical interventions |
| 1. AO | 2019 | Sam A, Currie K, Oh H, Flores-Mir C, Lagravére-Vich M. Reliability of different three-dimensional cephalometric landmarks in cone-beam computed tomography: A systematic review. Angle Orthod. 2019 Mar;89(2):317-332. doi: 10.2319/042018-302.1. Epub 2018 Nov 13. | not an orthodontic intervention |
| 1. AO | 2019 | Bastos RTDRM, Blagitz MN, Aragón MLSC, Maia LC, Normando D.Periodontal side effects of rapid and slow maxillary expansion: A systematic review. Angle Orthod. 2019 Jul;89(4):651-660. doi: 10.2319/060218-419.1. Epub 2019 Feb 11. | assessed exclusively adverse effects |
| 1. AO | 2018 | Ponce-Garcia C, Lagravere-Vich M, Cevidanes LHS, de Olivera Ruellas AC, Carey J, Flores-Mir C. Reliability of three-dimensional anterior cranial base superimposition methods for assessment of overall hard tissue changes: A systematic review. Angle Orthod. 2018 Mar;88(2):233-245. doi: 10.2319/071217-468.1. | not an orthodontic intervention |
| 1. AO | 2018 | Lima IFP, de Andrade Vieira W, de Macedo Bernardino Í, Costa PA, Lima APB, Pithon MM, Paranhos LR. Influence of reminder therapy for controlling bacterial plaque in patients undergoing orthodontic treatment: A systematic review and meta-analysis. Angle Orthod. 2018 Jul;88(4):483-493. doi: 10.2319/111117-770.1. | not an orthodontic intervention |
| 1. AO | 2018 | Sun W, Xia K, Tang L, Liu C, Zou L, Liu J. Accuracy of panoramic radiography in diagnosing maxillary sinus-root relationship: A systematic review and meta-analysis.Angle Orthod. 2018 Nov;88(6):819-829. doi: 10.2319/022018-135.1. | not an orthodontic intervention |
| 1. AO | 2017 | Eslami E, Katz ES, Baghdady M, Abramovitch K, Masoud MI. Are three-dimensional airway evaluations obtained through computed and cone-beam computed tomography scans predictable from lateral cephalograms? A systematic review of evidence. Angle Orthod. 2017 Jan;87(1):159-167. doi: 10.2319/032516-243.1. | not an orthodontic intervention |
| 1. AO | 2017 | Yi J, Sun Y, Li Y, Li C, Li X, Zhao Z. Cone-beam computed tomography versus periapical radiograph for diagnosing external root resorption: A systematic review and meta-analysis. Angle Orthod. 2017 Mar;87(2):328-337. doi: 10.2319/061916-481.1. | not an orthodontic intervention |
| 1. AO | 2017 | Currie K, Sawchuk D, Saltaji H, Oh H, Flores-Mir C, Lagravere M. Posterior cranial base natural growth and development: A systematic review. Angle Orthod. 2017 Nov;87(6):897-910. doi: 10.2319/032717-218.1. | not an orthodontic intervention |
| 1. AO | 2016 | Pachêco-Pereira C, Abreu LG, Dick BD, De Luca Canto G, Paiva SM, Flores-Mir C. Patient satisfaction after orthodontic treatment combined with orthognathic surgery: A systematic review. Angle Orthod. 2016 May;86(3):495-508. doi: 10.2319/040615-227.1. | review included orthognathic surgical interventions |
| 1. AO | 2016 | Gong A, Li J, Wang Z, Li Y, Hu F, Li Q, Miao D, Wang L. Cranial base characteristics in anteroposterior malocclusions: A meta-analysis.Angle Orthod. 2016 Jul;86(4):668-80. doi: 10.2319/032315-186.1. | not an orthodontic intervention |
| 1. AO | 2016 | Maniewicz Wins S, Antonarakis GS, Kiliaridis S. Predictive factors of sagittal stability after treatment of Class II malocclusions. Angle Orthod. 2016 Nov;86(6):1033-1041. | not the effects of orthodontic interventions were assessed |
| 1. AO | 2016 | Aminoshariae A, Aminoshariae A, Valiathan M, Kulild JC. Association of genetic polymorphism and external apical root resorption. Angle Orthod. 2016 Nov;86(6):1042-1049. | assessed exclusively adverse effects |
| 1. AO | 2016 | Rossini G, Parrini S, Castroflorio T, Fortini A, Deregibus A, Debernardi CL. Children's perceptions of smile esthetics and their influence on social judgment. Angle Orthod. 2016 Nov;86(6):1050-1055. | not an orthodontic intervention |
| 1. AO | 2015 | Aljabaa A, McDonald F, Newton JT. A systematic review of randomized controlled trials of interventions to improve adherence among orthodontic patients aged 12 to 18. Angle Orthod. 2015 Mar;85(2):305-13. doi: 10.2319/031214-184.1. | not an orthodontic intervention |
| 1. AO | 2015 | Pachêco-Pereira C, De Luca Canto G, Major PW, Flores-Mir C. Variation of orthodontic treatment decision-making based on dental model type: A systematic review. Angle Orthod. 2015 May;85(3):501-9. doi: 10.2319/051214-343.1. | not the effects of orthodontic interventions were assessed |
| 1. AO | 2015 | Al-Jewair TS. Meta-analysis on the mandibular dimensions effects of the MARA appliance in patients with Class II malocclusions. Angle Orthod. 2015 Jul;85(4):706-14. doi: 10.2319/052814-378.1. | only one reviewer |
| 1. AO | 2014 | Grewal Bach GK, Torrealba Y, Lagravère MO. Orthodontic bonding to porcelain: a systematic review. Angle Orthod. 2014 May;84(3):555-60. doi: 10.2319/083013-636.1. | not the effects of orthodontic interventions were assessed |
| 1. AO | 2014 | Marquezan M, Mattos CT, Sant'Anna EF, de Souza MM, Maia LC. Does cortical thickness influence the primary stability of miniscrews?: A systematic review and meta-analysis. Angle Orthod. 2014 Nov;84(6):1093-103. doi: 10.2319/093013-716.1. | not the effects of orthodontic interventions were assessed |
| 1. AO | 2013 | Lione R, Franchi L, Cozza P. Does rapid maxillary expansion induce adverse effects in growing subjects? Angle Orthod. 2013 Jan;83(1):172-82. doi: 10.2319/041012-300.1. | assessed exclusively adverse effects |
| 1. AO | 2012 | Al-Anezi SA, Harradine NW. Quantifying plaque during orthodontic treatment:. Angle Orthod. 2012 Jul;82(4):748-53. doi: 10.2319/050111-312.1. | assessed exclusively adverse effects |
| 1. AO | 2012 | Luu NS, Nikolcheva LG, Retrouvey JM, Flores-Mir C, El-Bialy T, Carey JP, Major PW. Linear measurements using virtual study models. Angle Orthod. 2012 Nov;82(6):1098-106. doi: 10.2319/110311-681.1. | not an orthodontic intervention |
| 1. AO | 2012 | Saltaji H, Major MP, Altalibi M, Youssef M, Flores-Mir C. Long-term skeletal stability after maxillary advancement with distraction osteogenesis in cleft lip and palate patients. Angle Orthod. 2012 Nov;82(6):1115-22. doi: 10.2319/011212-27.1. | assessed exclusively patients with congenital anomalies |
| 1. AO | 2012 | Santiago RC, de Miranda Costa LF, Vitral RW, Fraga MR, Bolognese AM, Maia LC. Cervical vertebral maturation as a biologic indicator of skeletal maturity. Angle Orthod. 2012 Nov;82(6):1123-31. doi: 10.2319/103111-673.1. | not an orthodontic intervention |
| 1. AO | 2010 | Archambault A, Lacoursiere R, Badawi H, Major PW, Carey J, Flores-Mir C. Torque expression in stainless steel orthodontic brackets. A systematic review. Angle Orthod. 2010 Jan;80(1):201-10. doi: 10.2319/080508-352.1. | review of laboratory studies |
| 1. AO | 2010 | Xiaoting L, Yin T, Yangxi C. Interventions for pain during fixed orthodontic appliance therapy. A systematic review. Angle Orthod. 2010 Sep;80(5):925-32. doi: 10.2319/010410-10.1. | not the effects of orthodontic interventions were assessed |
| 1. AO | 2010 | Magalhães IB, Pereira LJ, Marques LS, Gameiro GH. The influence of malocclusion on masticatory performance. A systematic review. Angle Orthod. 2010 Sep;80(5):981-7. doi: 10.2319/011910-33.1. | not the effects of orthodontic interventions were assessed |
| 1. KJO | 2021 | Giudice AL, Rustico L, Longo M, Oteri G, Papadopoulos MA, Nucera R. Complications reported with the use of orthodontic miniscrews: A systematic review. Korean J Orthod. 2021 May 25;51(3):199-216. doi: 10.4041/kjod.2021.51.3.199. PMID: 33984227; PMCID: PMC8133901. | assessed exclusively adverse effects |
| 1. KJO | 2021 | Sivarajan S, Mani SA, John J, Fayed MMS, Kook YA, Wey MC. The global distribution of permanent canine hypodontia: A systematic review. Korean J Orthod. 2021 Jan 25;51(1):55-74. doi: 10.4041/kjod.2021.51.1.55. PMID: 33446621; PMCID: PMC7837799. | not the effects of orthodontic interventions were assessed |
| 1. KJO | 2018 | Savoldi F, Papoutsi A, Dianiskova S, Dalessandri D, Bonetti S, Tsoi JKH, Matinlinna JP, Paganelli C. Resistance to sliding in orthodontics: misconception or method error? A systematic review and a proposal of a test protocol.Korean J Orthod. 2018 Jul;48(4):268-280. doi: 10.4041/kjod.2018.48.4.268. | review of laboratory studies |
| 1. KJO | 2018 | Nowrin SA, Jaafar S, Ab Rahman N, Basri R, Alam MK, Shahid F. Association between genetic polymorphisms and external apical root resorption: A systematic review and meta-analysis.Korean J Orthod. 2018 Nov;48(6):395-404. doi: 10.4041/kjod.2018.48.6.395. | assessed exclusively adverse effects |
| 1. KJO | 2016 | Hong SB, Kusnoto B, Kim EJ, BeGole EA, Hwang HS, Lim HJ. Prognostic factors associated with the success rates of posterior orthodontic miniscrew implants: A subgroup meta-analysis. Korean J Orthod. 2016 Mar;46(2):111-26. doi: 10.4041/kjod.2016.46.2.111. | not the effects of orthodontic interventions were assessed |
| 1. KJO | 2016 | Sawchuk D, Currie K1 Vich ML, Palomo JM, Flores-Mir C. Diagnostic methods for assessing maxillary skeletal and dental transverse deficiencies: A systematic review. Korean J Orthod. 2016 Sep;46(5):331-42. doi: 10.4041/kjod.2016.46.5.331. | not the effects of orthodontic interventions were assessed |
| 1. KJO | 2015 | Alessandri-Bonetti G, Ippolito DR, Bartolucci ML, D'Antò V, Incerti-Parenti S. Cephalometric predictors of treatment outcome with mandibular advancement devices in adult patients with obstructive sleep apnea: a systematic review. Korean J Orthod. 2015 Nov;45(6):308-21. doi: 10.4041/kjod.2015.45.6.308. | not the effects of orthodontic interventions were assessed |
| 1. O&C | 2021 | Berry S, Javed F, Rossouw PE, Barmak AB, Kalogirou EM, Michelogiannakis D. Influence of thyroxine supplementation on orthodontically induced tooth movement and/or inflammatory root resorption: A systematic review. Orthod Craniofac Res. 2021 May;24(2):206-213. doi: 10.1111/ocr.12428. Epub 2020 Oct 18. PMID: 32991769. | review of animal studies |
| 1. O&C | 2021 | Roomaney IA, Chetty M. Sella turcica morphology in patients with genetic syndromes: A systematic review. Orthod Craniofac Res. 2021 May;24(2):194-205. doi: 10.1111/ocr.12426. Epub 2020 Sep 28. PMID: 32920986. | not the effects of orthodontcis were assessed |
| 1. O&C | 2021 | Ahn HW, Kim SJ, Baek SH. Miniplate-anchored maxillary protraction in adolescent patients with cleft lip and palate: A literature review of study design, type and protocol, and treatment outcomes. Orthod Craniofac Res. 2021 Mar;24 Suppl 1:21-30. doi: 10.1111/ocr.12446. Epub 2020 Dec 7. PMID: 33253469. | assessed exclusively patients with congenital anomalies |
| 1. O&C | 2021 | Kaklamanos EG, Makrygiannakis MA, Athanasiou AE. Could medications and biologic factors affect post-orthodontic tooth movement changes? A systematic review of animal studies. Orthod Craniofac Res. 2021 Feb;24(1):39-51. doi: 10.1111/ocr.12411. Epub 2020 Aug 5. PMID: 32654394. | review of animal studies |
| 1. O&C | 2021 | Marques FBC, de Lima LS, Oliveira PLE, Magno MB, Ferreira DMTP, de Castro ACR, Maciel JVB, Ruellas ACO, Maia LC. Are temporomandibular disorders associated with facial asymmetry? A systematic review and meta-analysis. Orthod Craniofac Res. 2021 Feb;24(1):1-16. doi: 10.1111/ocr.12404. Epub 2020 Jul 19. PMID: 32608091. | not the effects of orthodontcis were assessed |
| 1. O&C | 2020 | Pinheiro FHSL, Drummond RJ, Frota CM, Bartzela TN, Dos Santos PB. Comparison of early and conventional autogenous secondary alveolar bone graft in children with cleft lip and palate: A systematic review. Orthod Craniofac Res. 2020 Nov;23(4):385-397. doi: 10.1111/ocr.12394. Epub 2020 Jun 28. PMID: 32446283. | assessed exclusively patients with congenital anomalies |
| 1. O&C | 2020 | Xiao WL, Jia KN, Yu G, Zhao N. Association between forkhead box E1 polymorphisms and risk of non-syndromic cleft lip with or without cleft palate: A meta-analysis. Orthod Craniofac Res. 2020 May;23(2):151-159. doi: 10.1111/ocr.12366. Epub 2020 Feb 5. PMID: 31944555. | not the effects of orthodontics were assessed |
| 1. O&C | 2020 | Kaklamanos EG, Makrygiannakis MA, Athanasiou AE. Do analgesics used for the pain experienced after orthodontic procedures affect tooth movement rate? A systematic review based on animal studies. Orthod Craniofac Res. 2020 May;23(2):143-150. doi: 10.1111/ocr.12357. Epub 2019 Nov 9. PMID: 31705727. | review of animal studies |
| 1. O&C | 2020 | Elsten EECM, Caron CJJM, Dunaway DJ, Padwa BL, Forrest C, Koudstaal MJ. Dental anomalies in craniofacial microsomia: A systematic review. Orthod Craniofac Res. 2020 Feb;23(1):16-26. doi: 10.1111/ocr.12351. Epub 2019 Oct 28. PMID: 31608577; PMCID: PMC7003932. | not the effects of orthodontics were assessed |
| 1. O&C | 2020 | Wu Z, Zhang X, Li Z, Liu Y, Jin H, Chen Q, Guo J. A Bayesian network meta-analysis of orthopaedic treatment in Class III malocclusion: Maxillary protraction with skeletal anchorage or a rapid maxillary expander. Orthod Craniofac Res. 2020 Feb;23(1):1-15. doi: 10.1111/ocr.12339. Epub 2019 Sep 15. PMID: 31452316. | Bayesian network analysis was used |
| 1. O&C | 2019 | Fang X, Qi R, Liu C. Root resorption in orthodontic treatment with clear aligners: A systematic review and meta-analysis. Orthod Craniofac Res. 2019 Nov;22(4):259-269. doi: 10.1111/ocr.12337. Epub 2019 Aug 29. PMID: 31323701. | assessed exclusively adverse effects |
| 1. O&C | 2019 | Iliadi A, Koletsi D, Eliades T. Forces and moments generated by aligner-type appliances for orthodontic tooth movement: A systematic review and meta-analysis. Orthod Craniofac Res. 2019 Nov;22(4):248-258. doi: 10.1111/ocr.12333. Epub 2019 Jul 9. PMID: 31237410. | review of laboratory studies |
| 1. O&C | 2019 | Tarallo F, Chimenti C, Paiella G, Cordaro M, Tepedino M. Biomarkers in the gingival crevicular fluid used to detect root resorption in patients undergoing orthodontic treatment: A systematic review. Orthod Craniofac Res. 2019 Nov;22(4):236-247. doi: 10.1111/ocr.12329. Epub 2019 Jul 2. PMID: 31207100. | not the effects of orthodontic interventions were assessed |
| 1. O&C | 2019 | Tasios T, Papageorgiou SN, Papadopoulos MA, Tsapas A, Haidich AB. Prevention of orthodontic enamel demineralization: A systematic review with meta-analyses. Orthod Craniofac Res. 2019 Nov;22(4):225-235. doi: 10.1111/ocr.12322. Epub 2019 May 27. PMID: 31081584. | not the effects of orthodontic interventions were assessed |
| 1. O&C | 2018 | Javed F, Akram Z, Barillas AP, Kellesarian SV, Ahmed HB, Khan J, Almas K. Outcome of orthodontic palatal plate therapy for orofacial dysfunction in children with Down syndrome: A systematic review.Orthod Craniofac Res. 2018 Feb;21(1):20-26. doi: 10.1111/ocr.12211. | assessed exclusively patients with congenital anomalies |
| 1. O&C | 2018 | Papageorgiou SN, Xavier GM, Cobourne MT, Eliades T. Effect of orthodontic treatment on the subgingival microbiota: A systematic review and meta-analysis.Orthod Craniofac Res. 2018 Nov;21(4):175-185. doi: 10.1111/ocr.12237. | assessed exclusively adverse effects |
| 1. O&C | 2018 | Scariot R, Corso PFCL, Sebastiani AM, Vieira AR.The many faces of genetic contributions to temporomandibular joint disorder: An updated review. Orthod Craniofac Res. 2018 Nov;21(4):186-201. doi: 10.1111/ocr.12239. Epub 2018 Sep 11. | not an orthodontic intervention |
| 1. O&C | 2017 | Antonarakis GS, Palaska PK2 Suri S. Permanent tooth agenesis in individuals with non-syndromic Robin sequence: a systematic review and meta-analysis. Orthod Craniofac Res. 2017 Nov;20(4):216-226. doi: 10.1111/ocr.12204. | assessed exclusively patients with congenital anomalies |
| 1. O&C | 2016 | Altmann AS, Collares FM, Leitune VC, Samuel SM. The effect of antimicrobial agents on bond strength of orthodontic adhesives: a meta-analysis of in vitro studies. Orthod Craniofac Res. 2016 Feb;19(1):1-9. doi: 10.1111/ocr.12100. Epub 2015 Aug 10. | review of laboratory studies |
| 1. O&C | 2015 | Tee BC, Sun Z. Mandibular distraction osteogenesis assisted by cell-based tissue engineering: a systematic review. Orthod Craniofac Res. 2015 Apr;18 Suppl 1:39-49. doi: 10.1111/ocr.12087. (in suppl issue) | review included orthognathic surgical interventions |
| 1. O&C | 2015 | De Luca Canto G, Pachêco-Pereira C, Lagravere MO, Flores-Mir C, Major PW. Intra-arch dimensional measurement validity of laser-scanned digital dental models compared with the original plaster models: a systematic review. Orthod Craniofac Res. 2015 May;18(2):65-76. doi: 10.1111/ocr.12068. | not an orthodontic intervention |
| 1. O&C | 2015 | Austin SL, Mattick CR, Waterhouse PJ. Distraction osteogenesis versus orthognathic surgery for the treatment of maxillary hypoplasia in cleft lip and palate patients: a systematic review.  Orthod Craniofac Res. 2015 May;18(2):96-108. doi: 10.1111/ocr.12063. | review included orthognathic surgical interventions |
| 1. O&C | 2014 | Koretsi V, Chatzigianni A, Sidiropoulou S. Enamel roughness and incidence of caries after interproximal enamel reduction: a systematic review. Orthod Craniofac Res. 2014 Feb;17(1):1-13. doi: 10.1111/ocr.12030. | not an orthodontic intervention |
| 1. O&C | 2014 | Pittayapat P, Limchaichana-Bolstad N, Willems G, Jacobs R. Three-dimensional cephalometric analysis in orthodontics: a systematic review. Orthod Craniofac Res. 2014 May;17(2):69-91. doi: 10.1111/ocr.12034. | not an orthodontic intervention |
| 1. O&C | 2013 | Perinetti G, Primožič J, Castaldo A, Di Lenarda R, Contardo L. Is gingival crevicular fluid volume sensitive to orthodontic tooth movement? A systematic review of split-mouth longitudinal studies. Orthod Craniofac Res. 2013 Feb;16(1):1-19. doi: 10.1111/ocr.12005. | assessed exclusively adverse effects |
| 1. O&C | 2013 | Andrade DC, Loureiro CA, Araújo VE, Riera R, Atallah AN. Treatment for agenesis of maxillary lateral incisors: a systematic review. Orthod Craniofac Res. 2013 Aug;16(3):129-36. doi: 10.1111/ocr.12015. | empty review |
| 1. O&C | 2012 | Angelopoulou MV, Vlachou V, Halazonetis DJ. Pharmacological management of pain during orthodontic treatment: a meta-analysis. Orthod Craniofac Res. 2012 May;15(2):71-83. doi: 10.1111/j.1601-6343.2012.01542.x. | assessed exclusively adverse effects |
| 1. O&C | 2012 | Gritsch K, Laroche N, Morgon L, Al-Hity R, Vico L, Colon P, Grosgogeat B. A systematic review of methods for tissue analysis in animal studies on orthodontic mini-implants. Orthod Craniofac Res. 2012 Aug;15(3):135-47. doi: 10.1111/j.1601-6343.2012.01548.x. | review of animal studies |
| 1. O&C | 2012 | Papadopoulos MA, Koumpridou EN, Vakalis ML, Papageorgiou SN. Effectiveness of pre-surgical infant orthopedic treatment for cleft lip and palate patients: a systematic review and meta-analysis. Orthod Craniofac Res. 2012 Nov;15(4):207-36. doi: 10.1111/j.1601-6343.2012.01552.x. | assessed exclusively patients with congenital anomalies |
| 1. O&C | 2011 | Fleming PS, Marinho V, Johal A. Orthodontic measurements on digital study models compared with plaster models: a systematic review. Orthod Craniofac Res. 2011 Feb;14(1):1-16. doi: 10.1111/j.1601-6343.2010.01503.x. | not an orthodontic intervention |
| 1. O&C | 2010 | Joss-Vassalli I, Grebenstein C, Topouzelis N, Sculean A, Katsaros C. Orthodontic therapy and gingival recession: a systematic review. Orthod Craniofac Res. 2010 Aug;13(3):127-41. doi: 10.1111/j.1601-6343.2010.01491.x. | assessed exclusively adverse effects |

*Description of the abbreviated journals:

Cochrane library: Cochrane Database of Systematic Reviews

AJODO: American Journal of Orthodontics and Dentofacial Orthopedics

EJO: European Journal of Orthodontics

AO: Angle Orthodontist

KJO: Korean Journal of Orthodontics

O&CR: Orthodontics and Craniofacial Research

**Excluded studies during the full text screening (n=45) with the rationale for exclusion**

| **Journal*** | **Year** | **Reference** | **Rationale for exclusion** |
| --- | --- | --- | --- |
| 1. Cochrane library | 2017 | Parkin N, Benson PE, Thind B, Shah A, Khalil I, Ghafoor S. Open versus closed surgical exposure of canine teeth that are displaced in the roof of the mouth.Cochrane Database Syst Rev. 2017 Aug 21;8:CD006966. doi: 10.1002/14651858.CD006966.pub3. | not the effects of orthodontic interventions were assessed |
| 1. Cochrane library | 2016 | Littlewood SJ, Millett DT, Doubleday B, Bearn DR, Worthington HV. Retention procedures for stabilising tooth position after treatment with orthodontic braces. Cochrane Database Syst Rev. 2016 Jan 29;(1):CD002283. doi: 10.1002/14651858.CD002283.pub4. | assessed exclusively adverse effects |
| 1. EJO | 2020 | Rekhi U, Catunda RQ, Gibson MP. Surgically accelerated orthodontic techniques and periodontal response: a systematic review. Eur J Orthod. 2020 Jan 15:cjz103. doi: 10.1093/ejo/cjz103. Epub ahead of print. PMID: 31942984. | the review is about a specific outcome of an intervention, which is ambiguous and could also be an adverse effect |
| 1. EJO | 2020 | Bortolotti F, Solidoro L, Bartolucci ML, Incerti Parenti S, Paganelli C, Alessandri-Bonetti G. Skeletal and dental effects of surgically assisted rapid palatal expansion: a systematic review of randomized controlled trials. Eur J Orthod. 2020 Sep 11;42(4):434-440. doi: 10.1093/ejo/cjz057. PMID: 31365925. | review included orthognathic surgical interventions |
| 1. EJO | 2020 | Papageorgiou SN, Koletsi D, Iliadi A, Peltomaki T, Eliades T. Treatment outcome with orthodontic aligners and fixed appliances: a systematic review with meta-analyses. Eur J Orthod. 2020 Jun 23;42(3):331-343. doi: 10.1093/ejo/cjz094. PMID: 31758191. | review included orthognathic surgical interventions |
| 1. EJO | 2019 | Bellini-Pereira SA, Pupulim DC, Aliaga-Del Castillo A, Henriques JFC, Janson G. Time of maxillary molar distalization with non-compliance intraoral distalizing appliances: a meta-analysis. Eur J Orthod. 2019 Nov 15;41(6):652-660. doi: 10.1093/ejo/cjz030. PMID: 31107942. | the review is about a specific outcome of an intervention, which is ambiguous and could also be an adverse effect |
| 1. EJO | 2019 | Swidi AJ, Griffin AE, Buschang PH. Mandibular alignment changes after full-fixed orthodontic treatment: a systematic review and meta-analysis. Eur J Orthod. 2019 Nov 15;41(6):609-621. Doi: 10.1093/ejo/cjz004. PMID: 30788505. | assessed exclusively adverse effects |
| 1. EJO | 2019 | Phuong A, Fagundes NCF, Abtahi S, Roberts MR, Major PW, Flores-Mir C. Additional appointments and discomfort associated with compliance-free fixed Class II corrector treatment: a systematic review. Eur J Orthod. 2019 Aug 8;41(4):404-414. Doi: 10.1093/ejo/cjy074. | assessed exclusively adverse effects |
| 1. EJO | 2019 | Almuzian M, Rizk MZ, Ulhaq A, Alharbi F, Alomari S, Mohammed H. Effectiveness of different debonding techniques and adjunctive methods on pain and discomfort perception during debonding fixed orthodontic appliances: a systematic review. Eur J Orthod. 2019 Sep 21;41(5):486-494. doi: 10.1093/ejo/cjz013. PMID: 30934051. | not an orthodontic intervention review |
| 1. EJO | 2018 | Cassina C, Papageorgiou SN, Eliades T. Open versus closed surgical exposure for permanent impacted canines: a systematic review and meta-analyses.Eur J Orthod. 2018 Jan 23;40(1):1-10. doi: 10.1093/ejo/cjx047. | not the effects of orthodontic interventions were assessed |
| 1. EJO | 2018 | Sampaziotis D, Tsolakis IA, Bitsanis E, Tsolakis AI. Open versus closed surgical exposure of palatally impacted maxillary canines: comparison of the different treatment outcomes-a systematic review.Eur J Orthod. 2018 Jan 23;40(1):11-22. doi: 10.1093/ejo/cjw077. | not the effects of orthodontic interventions were assessed |
| 1. EJO | 2018 | Papageorgiou SN, Papadelli AA, Eliades T. Effect of orthodontic treatment on periodontal clinical attachment: a systematic review and meta-analysis.Eur J Orthod. 2018 Apr 6;40(2):176-194. doi: 10.1093/ejo/cjx052. | assessed exclusively adverse effects |
| 1. EJO | 2017 | Buzatta LN, Shimizu RH, Shimizu IA, Pachêco-Pereira C, Flores-Mir C, Taba M Jr, Porporatti AL, De Luca Canto G. Gingival condition associated with two types of orthodontic fixed retainers: a meta-analysis. Eur J Orthod. 2017 Aug 1;39(4):446-452. doi: 10.1093/ejo/cjw057. | assessed exclusively adverse effects |
| 1. EJO | 2017 | Buck LM, Dalci O, Darendeliler MA, Papageorgiou SN, Papadopoulou AK. Volumetric upper airway changes after rapid maxillary expansion: a systematic review and meta-analysis. Eur J Orthod. 2017 Oct 1;39(5):463-473. doi: 10.1093/ejo/cjw048. | review included orthognathic surgical interventions |
| 1. EJO | 2016 | Bock NC, von Bremen J, Ruf S. Stability of Class II fixed functional appliance therapy--a systematic review and meta-analysis. Eur J Orthod. 2016 Apr;38(2):129-39. doi: 10.1093/ejo/cjv009. | assessed exclusively adverse effects |
| 1. EJO | 2016 | Sollenius O, Petrén S, Björnsson L, Norlund A, Bondemark L. Health economic evaluations in orthodontics: a systematic review. Eur J Orthod. 2016 Jun;38(3):259-65. doi: 10.1093/ejo/cjv040. | review did not assess the effect of a specific type of intervention(s), but assessed an undefined orthodontic intervention, e.g., orthodontic treatment as a whole |
| 1. EJO | 2016 | Janson G, Mendes LM, Junqueira CH, Garib DG. Soft-tissue changes in Class II malocclusion patients treated with extractions: a systematic review. Eur J Orthod. 2016 Dec;38(6):631-637. | review included orthognathic surgical interventions |
| 1. EJO | 2015 | Rossini G, Parrini S, Castroflorio T, Deregibus A, Debernardi CL. Periodontal health during clear aligners treatment: a systematic review. Eur J Orthod. 2015 Oct;37(5):539-43. doi: 10.1093/ejo/cju083. | assessed exclusively adverse effects |
| 1. EJO | 2014 | Yepes E, Quintero P, Rueda ZV, Pedroza A. Optimal force for maxillary protraction facemask therapy in the early treatment of class III malocclusion. Eur J Orthod. 2014 Oct;36(5):586-94. doi: 10.1093/ejo/cjt091. | not the effects of orthodontic interventions were assessed |
| 1. AJODO | 2020 | Pacha MM, Fleming PS, Johal A. Complications, impacts, and success rates of different approaches to treatment of Class II malocclusion in adolescents: A systematic review and meta-analysis. Am J Orthod Dentofacial Orthop. 2020 Oct;158(4):477-494.e7. doi: 10.1016/j.ajodo.2020.03.021. Epub 2020 Sep 2. PMID: 32888735. | assessed exclusively adverse effects |
| 1. AJODO | 2020 | Vandersluis YR, Suri S. Infective endocarditis and orthodontic implications in children: A review of the literature. Am J Orthod Dentofacial Orthop. 2020 Jan;157(1):19-28. doi: 10.1016/j.ajodo.2019.03.027. PMID: 31901273. | not the effects of orthodontic interventions were assessed |
| 1. AJODO | 2017 | Javidi H, Vettore M, Benson PE. Does orthodontic treatment before the age of 18 years improve oral health-related quality of life? A systematic review and meta-analysis. Am J Orthod Dentofacial Orthop. 2017 Apr;151(4):644-655. doi: 10.1016/j.ajodo.2016.12.011. | review did not assess the effect of a specific type of intervention(s), but assessed an undefined orthodontic intervention, e.g., orthodontic treatment as a whole |
| 1. AJODO | 2016 | Tsichlaki A, Chin SY, Pandis N, Fleming PS. How long does treatment with fixed orthodontic appliances last? A systematic review. Am J Orthod Dentofacial Orthop. 2016 Mar;149(3):308-18. doi: 10.1016/j.ajodo.2015.09.020. | review did not assess the effect of a specific type of intervention(s), but assessed an undefined orthodontic intervention, e.g., orthodontic treatment as a whole |
| 1. AJODO | 2015 | Pachêco-Pereira C, Pereira JR, Dick BD, Perez A, Flores-Mir C. Factors associated with patient and parent satisfaction after orthodontic treatment: a systematic review. Am J Orthod Dentofacial Orthop. 2015 Oct;148(4):652-9. doi: 10.1016/j.ajodo.2015.04.039. | review did not assess the effect of a specific type of intervention(s), but assessed an undefined orthodontic intervention, e.g., orthodontic treatment as a whole |
| 1. AJODO | 2014 | Mai W, He J, Meng H, Jiang Y, Huang C, Li M, Yuan K, Kang N. Comparison of vacuum-formed and Hawley retainers: a systematic review. Am J Orthod Dentofacial Orthop. 2014 Jun;145(6):720-7. doi: 10.1016/j.ajodo.2014.01.019. | assessed exclusively adverse effects |
| 1. AJODO | 2011 | Greenlee GM, Huang GJ, Chen SS, Chen J, Koepsell T, Hujoel P. Stability of treatment for anterior open-bite malocclusion: a meta-analysis. Am J Orthod Dentofacial Orthop. 2011 Feb;139(2):154-69. doi: 10.1016/j.ajodo.2010.10.019. | review included orthognathic surgical interventions |
| 1. AJODO | 2010 | Viglianisi A. Effects of lingual arch used as space maintainer on mandibular arch dimension: a systematic review. Am J Orthod Dentofacial Orthop. 2010 Oct;138(4):382.e1-4; discussion 382-3. doi: 10.1016/j.ajodo.2010.02.026. | review was conducted by one operator only |
| 1. AO | 2021 | Santana LG, Marques LS. Do adjunctive interventions in patients undergoing rapid maxillary expansion increase the treatment effectiveness? Angle Orthod. 2021 Jan 1;91(1):119-128. doi: 10.2319/051320-431.1. PMID: 33289794; PMCID: PMC8032281. | review included orthognathic surgical interventions |
| 1. AO | 2020 | Mecenas P, Espinosa DG, Cardoso PC, Normando D. Stainless steel or titanium mini-implants? Angle Orthod. 2020 Jul 1;90(4):587-597. doi: 10.2319/081619-536.1. PMID: 33378494; PMCID: PMC8028470. | not the effects of orthodontic interventions were assessed |
| 1. AO | 2020 | Moda LB, da Silva Barros ALC, Fagundes NCF, Normando D, Maia LC, Mendes SMDA. Lower fixed retainers: bonded on all teeth or only on canines? A systematic review. Angle Orthod. 2020 Jan;90(1):125-143. doi: 10.2319/013019-63.1. Epub 2019 Sep 19. PMID: 31536378; PMCID: PMC8087051. | assessed exclusively adverse effects |
| 1. AO | 2019 | Alakttash AM, Fawzi M, Bearn D. Adhesive precoated bracket systems and operator coated bracket systems: Is there any difference? A systematic review and meta-analysis. Angle Orthod. 2019 May;89(3):495-504. doi: 10.2319/051818-373.1. Epub 2018 Dec 17. | assessed exclusively adverse effects |
| 1. AO | 2017 | Diar-Bakirly S, Feres MF, Saltaji H, Flores-Mir C, El-Bialy T. Effectiveness of the transpalatal arch in controlling orthodontic anchorage in maxillary premolar extraction cases: A systematic review and meta-analysis.Angle Orthod. 2017 Jan;87(1):147-158. doi: 10.2319/021216-120.1. | assessed exclusively adverse effects |
| 1. AO | 2017 | Almasoud NN. Extraction of primary canines for interceptive orthodontic treatment of palatally displaced permanent canines: A systematic review. Angle Orthod. 2017 Nov;87(6):878-885. doi: 10.2319/021417-105.1. | not the effects of orthodontic interventions were assessed |
| 1. AO | 2015 | Andiappan M, Gao W, Bernabé E, Kandala NB, Donaldson AN. Malocclusion, orthodontic treatment, and the Oral Health Impact Profile (OHIP-14): Systematic review and meta-analysis. Angle Orthod. 2015 May;85(3):493-500. doi: 10.2319/051414-348.1. | review included orthognathic surgical interventions |
| 1. AO | 2013 | Long H, Zhou Y, Pyakurel U, Liao L, Jian F, Xue J, Ye N, Yang X, Wang Y, Lai W. Comparison of adverse effects between lingual and labial orthodontic treatment. Angle Orthod. 2013 Nov;83(6):1066-73. doi: 10.2319/010113-2.1. | assessed exclusively adverse effects |
| 1. AO | 2011 | Janson G, Branco NC, Fernandes TM, Sathler R, Garib D, Lauris JR. Influence of orthodontic treatment, midline position, buccal corridor and smile arc on smile attractiveness. Angle Orthod. 2011 Jan;81(1):153-61. doi: 10.2319/040710-195.1. | review did not assess the effect of a specific type of intervention(s), but assessed an undefined orthodontic intervention, e.g., orthodontic treatment as a whole |
| 1. KJO | 2017 | Papageorgiou SN, Höchli D, Eliades T. Outcomes of comprehensive fixed appliance orthodontic treatment: A systematic review with meta-analysis and methodological overview. Korean J Orthod. 2017 Nov;47(6):401-413. doi: 10.4041/kjod.2017.47.6.401. | review did not assess the effect of a specific type of intervention(s), but assessed an undefined orthodontic intervention, e.g., orthodontic treatment as a whole |
| 1. O&CR | 2021 | Lee DW, Park JH, Bay RC, Choi SK, Chae JM. Cortical bone thickness and bone density effects on miniscrew success rates: A systematic review and meta-analysis. Orthod Craniofac Res. 2021 Mar;24 Suppl 1:92-102. doi: 10.1111/ocr.12453. Epub 2020 Dec 16. | not the effects of orthodontic interventions were assessed |
| 1. O&CR | 2021 | Lee DW, Park JH, Bay RC, Choi SK, Chae JM. Cortical bone thickness and bone density effects on miniscrew success rates: A systematic review and meta-analysis. Orthod Craniofac Res. 2021 Mar;24 Suppl 1:92-102. doi: 10.1111/ocr.12453. Epub 2020 Dec 16. | assessed exclusively adverse effects |
| 1. O&CR | 2021 | Lee DW, Park JH, Bay RC, Choi SK, Chae JM. Cortical bone thickness and bone density effects on miniscrew success rates: A systematic review and meta-analysis. Orthod Craniofac Res. 2021 Mar;24 Suppl 1:92-102. doi: 10.1111/ocr.12453. Epub 2020 Dec 16. | not the effects of orthodontic interventions were assessed |
| 1. O&CR | 2021 | Copello FM, Marañón-Vásquez GA, Brunetto DP, Caldas LD, Masterson D, Maia LC, Sant'Anna EF. Is the buccal alveolar bone less affected by mini-implant assisted rapid palatal expansion than by conventional rapid palatal expansion?-A systematic review and meta-analysis. Orthod Craniofac Res. 2020 Aug;23(3):237-249. doi: 10.1111/ocr.12374. Epub 2020 Apr 16. PMID: 32187843. | assessed exclusively adverse effects |
| 1. O&CR | 2019 | Allen RK, Edelmann AR, Abdulmajeed A, Bencharit S. Salivary protein biomarkers associated with orthodontic tooth movement: A systematic review. Orthod Craniofac Res. 2019 May;22 Suppl 1:14-20. doi: 10.1111/ocr.12258. | not the effects of orthodontic interventions were assessed |
| 1. O&CR | 2018 | Cannavale R, Chiodini P, Perillo L, Piancino MG. Rapid palatal expansion (RPE): Meta-analysis of long-term effects.Orthod Craniofac Res. 2018 Nov;21(4):225-235. doi: 10.1111/ocr.12244. | assessed exclusively adverse effects |
| 1. O&CR | 2015 | Al-Saleh MAQ, Alsufyani N, Flores-Mir C, Nebbe B, Major PW. Changes in temporomandibular joint morphology in class II patients treated with fixed mandibular repositioning and evaluated through 3D imaging: a systematic review. Orthod Craniofac Res. 2015 Nov;18(4):185-201. doi: 10.1111/ocr.12099. | the review is about a specific outcome of an intervention, which is ambiguous and could also be an adverse effect |
| 1. O&CR | 2011 | von Bremen J, Ruf S. Orthodontic and dentofacial orthopedic management of juvenile idiopathic arthritis: a systematic review of the literature. Orthod Craniofac Res. 2011 Aug;14(3):107-15. doi: 10.1111/j.1601-6343.2011.01514.x. | review included orthognathic surgical interventions |

*Description of the abbreviated journals:

Cochrane library: Cochrane Database of Systematic Reviews

AJODO: American Journal of Orthodontics and Dentofacial Orthopedics

EJO: European Journal of Orthodontics

AO: Angle Orthodontist

KJO: Korean Journal of Orthodontics

O&CR: Orthodontics and Craniofacial Research
